# ICOS deficiency hampers the homeostasis, development and function of NK cells

**DOI:** 10.1371/journal.pone.0219449

**Published:** 2019-07-08

**Authors:** María Montes-Casado, Gloria Ojeda, Laura Aragoneses-Fenoll, Daniel López, Belén de Andrés, María Luisa Gaspar, Umberto Dianzani, José M. Rojo, Pilar Portolés

**Affiliations:** 1 Centro Nacional de Microbiología, Instituto de Salud Carlos III (ISCIII), Majadahonda, Madrid, Spain; 2 Interdisciplinary Research Center of Autoimmune Diseases (IRCAD) and Department of Health Sciences, University of Piemonte Orientale (UPO), Novara, Italy; 3 Centro de Investigaciones Biológicas, Consejo Superior de Investigaciones Científicas (CSIC), Madrid, Spain; University of Colorado Denver School of Medicine, UNITED STATES

## Abstract

Signaling through the inducible costimulator ICOS is required for the homeostasis and function of various immune cell populations, with an outstanding role in the generation and maintenance of germinal centers. Very recently, it has been suggested that the clinical phenotype of ICOS-deficient patients is much broader than initially anticipated and the innate immune response might be also affected. However, the role of the ICOS/ICOS-Ligand axis in the homeostasis and development of innate NK cells is not known, and reports on its participation in NK cell activation are scarce. NK cells may express low levels of ICOS that are markedly enhanced upon activation. We show here that ICOS-deficient (ICOS-KO) mice present low NK cell numbers and defects in the homeostasis of these cells, with delayed maturation and altered expression of the developmental NK cell markers CD122, NK1.1, CD11b or CD27. Our experiments in mixed bone marrow chimera mice indicate that, both, cell-intrinsic defects of ICOS-KO NK and deficiencies in the milieu of these mice contribute to the altered phenotype. ICOS-deficient NK cells show impaired production of IFN-γ and cytotoxicity, and a final outcome of defects in NK cell-mediated effector function during the response to poly(I:C) or vaccinia virus infection *in vivo*. Interestingly, we show that murine innate cells like IL-2-cultured NK and bone marrow-derived dendritic cells can simultaneously express ICOS and ICOS-Ligand; both molecules are functional in NK intracellular signaling, enhancing early phosphorylation of Akt and Erk, or IFN-γ secretion in IL-2-activated NK cells. Our study shows the functional importance of the ICOS/ICOS-L pair in NK cell homeostasis, differentiation and activity and suggests novel therapeutic targets for NK manipulation.

## Introduction

The inducible costimulator ICOS is up-regulated following T and natural killer (NK) cell activation whereas it is constitutively expressed in NKT cells, ILC2 cells, and in a small subpopulation of CD4^+^IL10^+^ T cells with regulatory functions [1–9]. ICOS and its ligand ICOS-L (also known as CD275, B7h, B7-H2) form a monogamous pair, and ligand expression is down-regulated by the expression of ICOS [10]. ICOS-L is expressed mainly, but not exclusively, by antigen presenting cells (APCs), and its interaction with ICOS is determinant for the differentiation and effector function of T helper cell subsets, including Th1, Th2, Th17, T follicular helper and CD4^+^Foxp3^+^ T regulatory cells [11–15]. Subsequently, ICOS has an outstanding role in germinal center formation, isotype class switching and development of memory B cells [12, 16–18]. Mutations in the ICOS gene is a cause of common variable immunodeficiency (CVID) in humans, which profoundly affects different aspects of T and B cell immune responses [12, 15]. Recently, an increased susceptibility to viral and opportunistic infections has been described in ICOS-deficient patients [19] thus suggesting that innate cells may be also affected in this combined immunodeficiency.

The ICOS/ICOS-L interaction plays an important role during the intrathymic development of CD3^+^NK1.1^+^ invariant NKTs (iNKT) [20, 21]. Interestingly, some innate immune cells (including murine ILC2 [5] and human monocyte-derived dendritic cells [22]) can simultaneously express ICOS and its ligand, and indeed, ICOS/ICOS-L signaling is essential to maintain their homeostasis and activity, and for their participation in the pathogenesis of immune diseases [5, 22]. However, the role of ICOS in the homeostasis and development of NK cells has not yet been reported.

NK cells play a critical role in the early response of the host to viruses, bacteria and tumor cells, thus serving as a link between the innate and the adaptive arms of immunity. Awareness of the importance of NK cells in immune responses has been further increased by recent data describing adaptive immunity characteristics, including memory, in NK cells [23] or their capacity to modulate adaptive immune cells [24]. Therefore, abnormalities in NK cells development, homeostasis or functionality may have serious consequences to the immune response.

NK cells are part of Group 1 within the functionally heterogeneous cell populations classified as innate lymphoid cells (ILCs). ILCs lack clonotypic antigen receptors and they are developmentally independent of recombination activating gene (RAG) proteins, yet they are derived from the common lymphoid progenitor (CLP) which gives also rise to the precursors of B and T lymphocytes [24, 25]. Group 1 ILCs produce Th1-associated cytokines like IFN-γ, and include NK cells and ILC1s. Both cell types express the markers NKp46 and NK1.1 (in the C57Bl/6 mouse) and the T-box transcription factor T-bet but differ in the expression of adhesion molecules as CD49a, b, d or CD62L. Besides, the development and maturation of NK cells requires Eomesodermin (Eomes) [26, 27].

The early stages of NK cell development occur primarily in the bone marrow (BM), although the process is not completely understood. The NK cell precursors (NKP) express CD122 (IL-2/15-Receptor β chain), IL-7Rα and lack common lineage markers including the NK cell markers NK1.1 and CD49b (DX5, VLA-2, α2 integrin) and some ILC markers including CD49a (VLA-1, α1 integrin), CD49d (VLA-4, α4 integrin), IL33-Receptor and the transcription factor RORγT [25, 27–29]. NKPs give rise to immature NK (iNK) cells that express NK1.1 and T-bet, but lose IL-7Rα expression. Progression of iNK into the mature phenotype is marked by expression of NKp46, CD49b and Eomes [25, 30]. The expression of inhibitory and activating receptors drives the *“education”* or *“licensing”* of NK cells, and their acquisition of functional competence (i.e.: cytotoxicity and IFN-γ production), allowing these cells to egress from the BM as mature NK cells (mNK) [25, 26]. NK cells continue to differentiate in the periphery, progressively acquiring new phenotypic features and immune functions, enhancing CD11b or KLRG1 expression and cytokine production, and losing CD27 and TRAIL (tumor necrosis factor-related apoptosis-inducing ligand) [25]. A four-stage model defined by the surface markers CD27 and CD11b has been proposed for mouse NK cells progressive maturation [31]: CD11b^low^CD27^low^ (most immature); CD11b^low^CD27^high^; CD11b^high^CD27^high^; and CD11b^high^CD27^low^ (most mature). These stages are linked to the progressive acquisition of NK cell effector activity, including cytotoxicity and cytokine secretion [31].

NK cell activation and homeostasis require cytokines like IL-2, IL-15 and type I-IFN in among others. However, in spite of the expression of CD28-family members by these cells [6, 32, 33], including ICOS, little is known about the costimulatory requirements of NK cells and there are few reports addressing the role of ICOS for NK cell function [6, 34]. Accordingly, we have used ICOS-KO mice to assess the importance of ICOS in NK cell homeostasis and differentiation, and in the response to virus infection *in vivo*. In addition, we show that mouse IL-2-cultured NK and BM-derived dendritic cells (BMDCs) may co-express ICOS and ICOS-L, unlike the single expression of each molecule in T and B lymphocytes; and both the costimulator and ligand are able to signal NK cells. The consequences of ICOS and ICOS-L co-expression and their signaling capacity are discussed.

## Materials and methods

### Mice

The animals used in this study were C57BL/6J mice (WT), ICOS^-/-^ (ICOS-KO) mice on a C57BL/6J background (kindly provided by Dr. R.A. Flavell [35]), B6.SJL.CD45.1^+^ and Rag2^-/-^γc^-/-^ mice. Eight to fourteen week old sex-matched animals were used in these experiments.

The mice were bred and housed at the animal care facilities of the Centro de Investigaciones Biológicas (CSIC, Madrid, Spain) or at the Instituto de Salud Carlos III (Majadahonda, Madrid, Spain), under specific pathogen free conditions with sterilized food, water, bedding and environmental enrichment.

All experimental procedures were approved by the Ethics and Animal Welfare Committees of the Instituto de Salud Carlos III (OEBA-Majadahonda and CEIYBA) and that of the Consejo Superior de Investigaciones Científicas. The study was carried out under project licenses PROEX 330/15 (to PP), PROEX 181/15 (to JMR) and PROEX 110/15 (to BdA) approved by the Consejeria de Medio Ambiente y Ordenación del Territorio de la Comunidad de Madrid. All the studies involving animals have been conducted according to local, national and European Union guidelines.

### Antibodies and other reagents

The antibodies used in the flow-cytometry analysis were conjugated to APC, FITC, PE, PE-Cy7, APC-eFluor780, Brilliant Violet 421, Brilliant Violet 711, Brilliant Violet 786 or biotin, and they recognized the following mouse proteins: CD3 (145-2C11), CD11b (M1/70), CD11c (N418), CD19 (1D3), CD27 (LG.7F9), CD69 (H1.2F3), CD122 (IL-2Rβ chain; TM-Beta1), CD275 (ICOS-L; HK5.3), CD278 (ICOS; C398.4), granzyme B (16G6), NK1.1 (PK136), IL-33 receptor (RMST2-33), CD49d (LPAM, α4 integrin) (DATK32), RORγT (B2D) and IFN-γ (XMG1.2); these antibodies were from eBioscience Inc. (San Diego, CA, USA). Anti-CD28 (37.51), -CD49b (α2 integrin, DX5), -CD80 (16-10A1), and anti-CD86 (GL-1), were from BioLegend (San Diego, CA, USA). Anti-CD49a (α1 integrin, HMα1) was from Miltenyi Biotec (Berisch Gladbach, Germany). Isotype control antibodies were obtained from eBiosciences, and PE-, Brilliant Violet 711-, PECy7- and APC-eFluor780-Streptavidin were from Southern Biotech (Birmingham, AL, USA), BioLegend and eBiosciences, respectively. Finally, anti-STAT5 (pY694; 47/STAT5) and the appropriate isotype control were purchased from BD Biosciences (San Jose, CA, USA). Recombinant human IL-2 (hIL-2, Immunotools GmbH, Friesoythe, Germany), recombinant murine IL-15 (Miltenyi Biotech) and recombinant murine GM-CSF (Peprotech, Rocky Hill, NJ, USA) were used.

The following purified antibodies were used to activate NK cells: anti-NK1.1 (PK136), anti-CD278 (anti-ICOS; clone 7E.17G9 and clone C398.4A), anti-CD275 (anti-ICOS-L; HK5.3) with their appropriate controls were from eBioscience. Recombinant mouse B7-H2 (ICOS-L)-human Fc from R&D Systems (Minneapolis, MN, USA) and mouse ICOS-human Fc fusion proteins from [36] or R&D Systems were also used. Anti-CD16/32 (anti-Fcγ RIII/II, Clone 93; BioLegend) was used to block the Fc receptor. All the antibodies used for cell activation assays were ultra-LEAF (low endotoxin, azide free) affinity chromatography-purified preparations.

The rabbit antiserum specific to the dual phosphorylated Erk (anti-active MAPK; ref. V6671) was supplied by Promega (Madison, WI, USA); rabbit anti-phospho-P38 MAPK (Thr180/Tyr182; #9211) and anti-phospho-Akt (Ser473; #9271) antibodies were from Cell Signaling Technology (Leiden, The Netherlands). Rabbit polyclonal anti-ERK antibody was from Upstate Biotechnology Inc. (Lake Placid, NY, USA) and the rabbit polyclonal anti-Akt and anti-P38 antibodies were from Santa Cruz Biotechnology.

Rabbit purified anti-asialo-GM1 Ig (Wako Chemicals GmbH; Neuss, Germany) was used for *in vivo* depletion of NK cells.

### Primary cells and cell lines

Primary cells obtained from spleen, BM or peritoneal exudate to be used in these experiments were suspended in complete culture medium (CC, Click's Medium [37] supplemented with 10% heat-inactivated fetal bovine serum (FCSi)). Red blood cells were lysed in erythrocyte lysis solution (Sigma-Aldrich; St Louis, MO, USA) and after washing, the cell suspensions were counted and adjusted to the concentration required for each experiment in CC medium or the appropriate buffer.

BM was obtained from the posterior limb bones, as described previously [38], and the cells were processed under sterile conditions and prepared similarly to the spleen cells.

The SR.D10 cell line (a subclone of the D10.G4.1 CD4^+^ T cell line) and the H4^-^A5 clone (an ICOS deficient mutant of SR.D10) [39] were used as controls for RT-qPCR. All cell lines were checked for mycoplasma contamination.

### Isolation and culture of NK cells

NK cells were isolated from the spleen cell suspensions by negative selection using the mouse "NK Cell Isolation Kit II" (Miltenyi Biotec), following the manufacturer's instructions. The purified NK cells were washed twice in CC medium and adjusted to the desired concentration in the same medium. When assessed by flow cytometry, these cells were routinely higher than 80% CD3^-^NK1.1^+^CD122^+^ NK cells, and no CD3^-^DX5^-^CD127^+^ ILC1 cells were detected.

Where indicated, the NK cell-enriched cell suspension was cultured at 1x10^6^ cells/ml in CC with 2,000 U/ml hIL-2 in round-bottom 96 well tissue culture plates (200 μl/well) for up to 7 days, replacing half of the medium with fresh hIL-2-containing CC medium every 2–3 days. From day 4 onwards, the NK cell cultures were routinely 98–99% pure and they were used until day 7.

### NK cell activation *in vitro*

The 96 well culture plates (Costar, Ref.3598, Corning Inc., Corning, NY, USA) were coated by overnight incubation at 4°C with 50 μl/well of the corresponding monoclonal antibody (mAb), fusion protein or isotype control in PBS. IgG2a isotype control, or Abs against ICOS-L, ICOS or recombinant ICOS-Fc or ICOS-L-Fc were used at 10 μg/ml; anti-NK1.1 (PK136) was used at 1 μg/ml. The wells were then washed three times with PBS and filled with CC medium. Cells previously blocked with 0.5 μg/ml anti-CD16/32 or purified normal mouse Ig were dispensed into the wells in a final volume of 200 μl and at a concentration of 10^6^ cells/ml. The plates were cultured (37°C, 5% CO_2_) for 24 hours in the presence of hIL-2 (2,000 U/ml); then, the cells and the supernatants were collected to be used in experiments or for ELISA analysis.

### Bone marrow-derived dendritic cells generation, culture and maturation

BMDCs were obtained following previously described methods [38, 40] with some modifications. Briefly, BM cells (3×10^6^/ml) were plated into 6-well tissue culture plates in culture medium supplemented with GM-CSF (50 ng/ml). After 48 h in culture at 37°C in an atmosphere of 5% CO_2_, two thirds of the medium were replaced with fresh medium supplemented with GM-CSF to remove non-adherent cells, leaving the anchored colonies of DCs. Two days later, additional GM-CSF supplemented medium was added and the resulting BMDCs (immature BMDCs) were collected after 5–8 days in culture. To obtain mature BMDCs, immature BMDCs were cultured *in vitro* for 24 h in the presence of LPS (100 ng/ml, Lipopolysaccharide from E. coli serotype R515, TLR grade, Enzo Biochem, Inc., Farmingdale, NY, USA) or Poly(I:C) (1 μg/ml; Invivo, San Diego, CA, USA). For experiments to quantify gene expression, CD11c^+^ or CD11c^+^CD86^+^CD80^high^ cells were sorted from the BMDCs using a FACS Aria I flow cytometer (BD Biosciences, San Jose, CA, USA).

### Flow cytometry

Single cell suspensions were incubated for 10 min with heat-inactivated normal mouse serum (10% in staining buffer) to block Fc receptors and then with the indicated labelled antibodies; after extensive washing they were analyzed by flow cytometry gating lymphoid cells according to FSC/SSC.

To better identify conventional NK cells, and to exclude ILC1, 2 and 3 from the analysis, samples were labelled with a mix of antibodies against surface molecules (anti-CD49a, -CD49d and -IL33-R biotinylated Abs) and intracellular RORγT (anti-RORγT-PE Ab). This mixture of Abs is referred to from now on as "ILC markers exclusion mix". Cells positive for those markers were electronically excluded, and the remaining cells are referred as ILC^-^ in the text. NK cells were thus selected as CD3^-^NK1.1^+^CD49a^-^CD49d^-^IL33R^-^RORγT^-^ (CD3^-^NK1.1^+^ILC^-^).

To determine cytokine-producing cells, the cells were stimulated with PMA (20 ng/ml) (Sigma-Aldrich) plus ionomycin (200 μg/ml) (Sigma-Aldrich) for 4 h, and brefeldin A (10 μg/ml, Sigma-Aldrich) was added for the last 3.5 h. After staining of cell surface markers, the cells were fixed and permeabilized with Cytofix/Cytoperm kit (BD Biosciences); then intracellular cytokines were stained.

Apoptosis was determined by flow cytometry using the Annexin V-FITC kit (eBioscience), according to the manufacturer’s instructions.

To stain phosphorylated STAT-5, BD Cytofix/Cytoperm (Fixation/Permeabilization Solution Kit, BD Biosciences) was used with the permeabilization buffer III for intranuclear signaling molecules. To detect granzymes or the RORγT transcription factor, the cells were fixed, permeabilized and stained as recommended in the FoxP3 detection kit (eBiosciences).

In all cases, data were acquired on FACSCanto II or LSRFortessa flow cytometers (BD Biosciences, San Jose, CA, USA) and analyzed with the BD FACSDIVA (BD Biosciences, San Jose, CA, USA) software.

### Immunoblotting

Phosphorylated Akt, Erk and P38 MAP kinases were determined in lysates of NK cells. NK cells purified from the spleen of WT mice were cultured for 4 days in the presence of hIL-2 and then washed with PBS and activated for 20 min at 37°C in serum-free medium by mixing the cells at 50x10^6^/ml with an equal number of latex beads (Polybead Polystyrene 4.5 Micron Microspheres (Polysciences Inc., Warrington, PA, USA) coated with anti-NK1.1, anti-ICOS-L, anti-ICOS or control antibodies (5 μg/ml). The cell lysates were obtained, separated by SDS-PAGE, transferred to PVDF membranes, and the immunoblots were probed as described previously in detail [41, 42]. ImageJ 1.51j8 (National Institutes of Health, Bethesda, MD, USA) was used for densitometry analysis of the immunoblots.

### Cytokine quantification

The cytokines in culture supernatants were assessed using "sandwich" ELISA (Ready-Set-Go ELISA and Platinum ELISA, eBioscience) according to the manufacturer's instructions.

### ICOS and ICOS-L mRNA expression

RNA was extracted using the mirVana miRNA isolation kit (Ambion-Life Technologies, Carlsbad, CA, USA), following the manufacturer's instructions. The cDNAs were generated from 1–5 μg of RNA using random hexamer primers and the ThermoScript RT-PCR System for First-Strand cDNA Synthesis (Invitrogen-Life Technologies, Carlsbad, CA, USA).

Gene expression was determined by real-time PCR using an iQ5 Real-Time PCR detection system (Bio-Rad Laboratories, Hercules, CA, USA) with the reactions including the iQ SYBR Green Supermix, 5 ng cDNA and 500 nM of each oligonucleotide primer, as follows: *TBP-F*: *5´-GGCGGTTTGGCTAGGTTT-3´; TBP-R*: *5´-GGGTTATCTTCACACACCATGA-3´; ICOS-F*: *5´-GCACTGGAGGAGAAGACTGC-3´; ICOS-R*: *5´-CCGAGCCATTGATTTCTCC-3´; ICOS-L-F*: *5´-CGCACCATGCAGCTAAAGT-3´; ICOS-L-R*: *5´-AAACATGGAGCTTCTTCCAAAC-3´*.

The data obtained from real-time PCR were normalized to TBP (TATA-binding protein) as an internal control and analyzed using the 2(-ΔΔ C(T)) Method [43] relative to the expression of each gene in WT cells (value 1).

### Generation of mixed bone marrow chimeras

Rag2^-/-^γc^-/-^ recipient mice were irradiated with 150 rad of total body irradiation from a ^137^Cs source and were i.v. injected with 10^7^ donor BM cells containing a 1:1 mixture of B6.SJL.CD45.1^+^ (WT CD45.1^+^) plus ICOS^−/−^ CD45.2^+^ BM cells. Sex-matched donor and recipients were used. The chimeric condition of the mice was assessed by flow cytometric analysis of blood cells every two weeks. Chimeras were sacrificed and used for analysis nine weeks after transplantation. Spleen cells of chimeric mice were stained using the reagents and techniques described previously in this section and with the same strategy of conventional NK gating.

### Response of NK cells to vaccinia virus *in vivo*

Female 8–10 week old mice were infected by i.p. inoculation of 10^6^ plaque forming units (pfu) of vaccinia virus VACV-WR. For NK depletion, the mice were intravenously injected (in the tail vein) with 50 μg of anti-Asialo-GM1 rabbit Ab (Wako Chemicals GmbH), 24 h before and 24 h after vaccinia virus inoculation. The mice were sacrificed 48 hours after infection, spleen cell suspension and peritoneal exudate cells (PEC) were obtained, and analyzed by flow cytometry. The viral load in the ovaries of these mice was assessed in a plaque-forming assay as described elsewhere [44], with minor modifications. In brief, female mice were sacrificed 48 h post-infection, and their ovaries were harvested and stored at −80°C in 0.5 ml of PBS. The ovaries from individual mice were homogenized, freeze-thawed over three cycles and sonicated. Serial dilutions of homogenates were plated onto confluent cultures of the vaccinia virus-susceptible monkey kidney cell line CV1. After 24 h in culture at 37°C, the plates were stained with crystal violet and the plaques were counted.

### Statistical analysis

Data were analyzed using the GraphPad Prism 7 software (GraphPad Prism Software Inc., La Jolla, CA, USA) and they are shown as the mean ± standard error of the mean (SEM). Data from biological replicates (mice) were analyzed individually. Direct group-group comparisons were assessed using Student´s t test for unpaired, two-tailed experimental design. In the statistical analysis of mixed BM chimeric mice, CD45.1 vs. CD45.2 cells within each mouse were compared by using the Wilcoxon matched pairs signed rank test. Asterisks indicate significant differences between adjacent bars or indicated pairs as follows: *p<0.05, **p<0.01, ***p<0.001, ****p<0.0001.

## Results

### ICOS-KO mice have fewer NK cells and their apoptosis is enhanced

**Fig 1:**
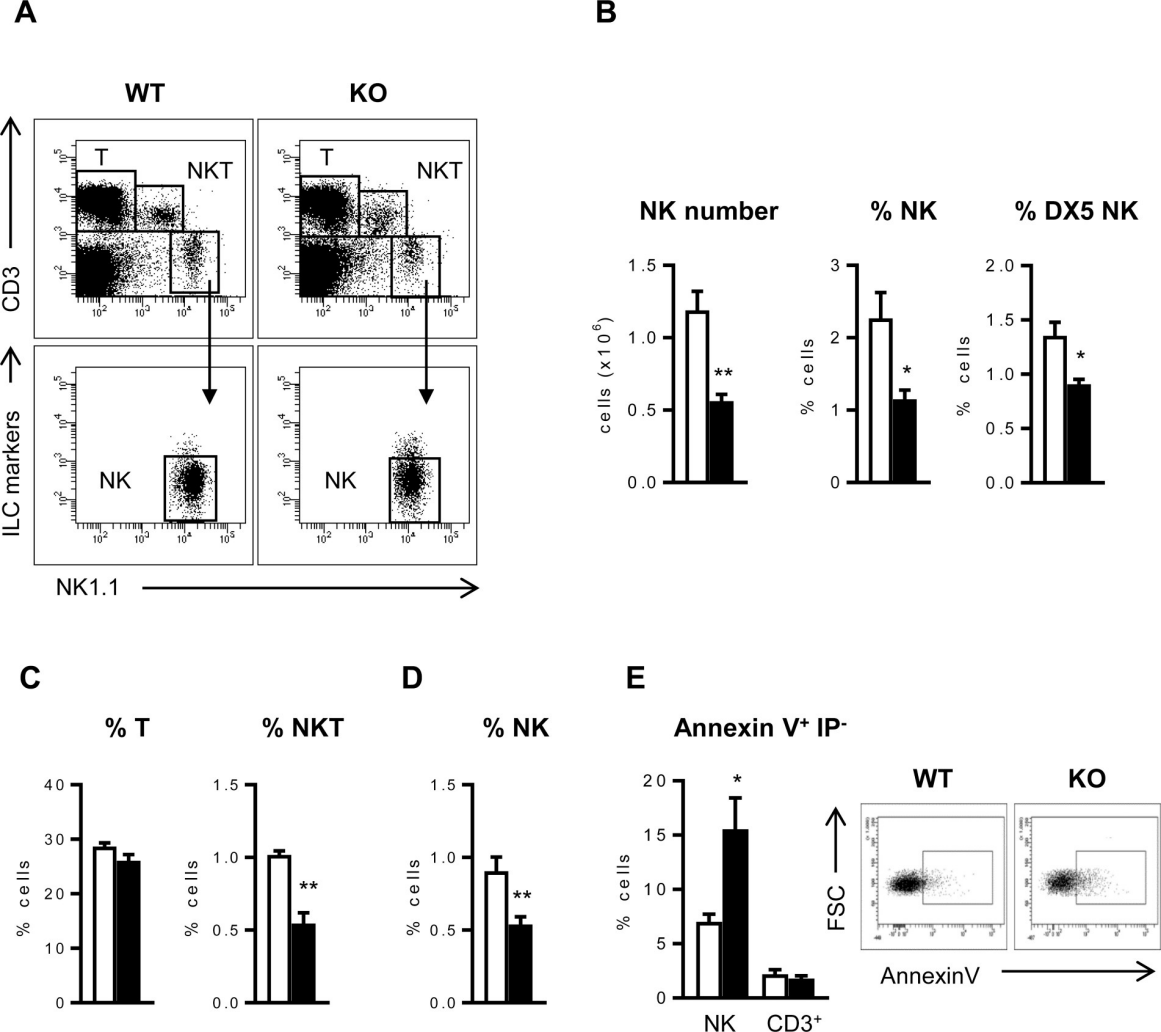
ICOS-deficiency is linked to a defect in NK cell number and survival. (A) Representative dot-plots showing the strategy to select different subpopulations (T, NKT and NK) in the spleen of WT and ICOS-KO mice. NK cells (NK) were gated as CD3^-^NK1.1^+^ILC^-^ to exclude ILC as indicated in Methods. (B) Analysis of the NK cell population in the spleen of WT and ICOS-KO mice. Cells were gated as CD3^-^NK1.1^+^ILC^-^ (NK) or CD3^-^DX5^+^ILC^-^ (DX5^+^). (C) Relative number of T (CD3^+^NK1.1^-^, left) and NKT (CD3^+^NK1.1^+^, right) subpopulations among the WT and ICOS-KO spleen cells. (D) Analysis of NK cells (CD3^-^NK1.1^+^ILC^-^) in BM of WT and ICOS-KO mice. (E) Left, early spontaneous apoptosis measured as proportion of Annexin V^+^PI^-^ cells in freshly obtained NK or CD3^+^ spleen cells from WT and ICOS-KO mice. Right, representative dot plots showing apoptosis in NK cells. Bars, WT (white) and ICOS-KO (black). A-E, representative dot-plots and mean±SEM are shown. N = 6 individual mice per group are shown in A to D, and N = 4 in E. *p<0.05, **p<0.01 between adjacent bars.

To assess the impact of ICOS-deficiency on NK cells, spleen cell populations in ICOS-KO and WT mice were compared. In the spleen of ICOS-KO mice, significantly fewer NK cells were detected, identified as CD3^-^NK1.1^+^CD49a^-^CD49d^-^IL33R^-^RORγT^-^ (CD3^-^NK1.1^+^ILC^-^, Fig 1A and 1B). Staining with anti-DX5 (CD49b), as an alternative NK cell marker, confirmed a smaller proportion of NK cells in ICOS-KO mice (Fig 1B). No significant differences in the total T lymphocytes were detected in ICOS-KO mice, although they had fewer CD3^+^NK1.1^+^ NKT cells (Fig 1C) and some T cell subpopulations, consistent with previous observations in mice lacking ICOS/ICOS-L interactions [11, 20, 21]. The reduced number of NK cells in the spleen of ICOS-KO mice could be due to their impaired development in the BM or to altered homeostasis in the periphery. Fewer NK cells were also found in the BM of ICOS-KO mice (Fig 1D), suggesting defective NK cell development in the ICOS-KO mice and prompting us to analyze ICOS-KO NK cells in both the BM and the periphery.

To check whether ICOS deficiency might also affect NK cell survival, spontaneous apoptosis was determined. Indeed, NK cells from the spleen of ICOS-KO mice had enhanced apoptosis when measured with Annexin V (Fig 1E). Enhanced apoptosis was not evident in other lymphoid populations like total CD3^+^ T cells (Fig 1E), although it was observed in minor subpopulations of T cells like natural Treg CD4^+^CD25^+^Foxp3^+^ cells [11].

Since NK cell homeostasis and activation is influenced by cell-to-cell interactions and cytokines like IL-2 or IL-15 [45–47], a deficient response to these interleukins might affect NK cell survival. However, NK cells purified from ICOS-KO mice and cultured in the presence of IL-2 or IL-15 did not show impaired proliferation *in vitro* when compared to cells from WT mice (S1A Fig). Similarly, phospho-STAT5, an intermediary of IL-2-mediated cell signaling [48], was unaltered (S1B Fig) indicating no apparent defects in IL-2 or IL-15 response in ICOS-KO purified NK cells *in vitro*. IL-2 induced robust ICOS expression in WT NK cells (S1C and S1D Fig) as expected.

### NK maturation in the bone marrow and spleen is altered in ICOS-KO mice

**Fig 2:**
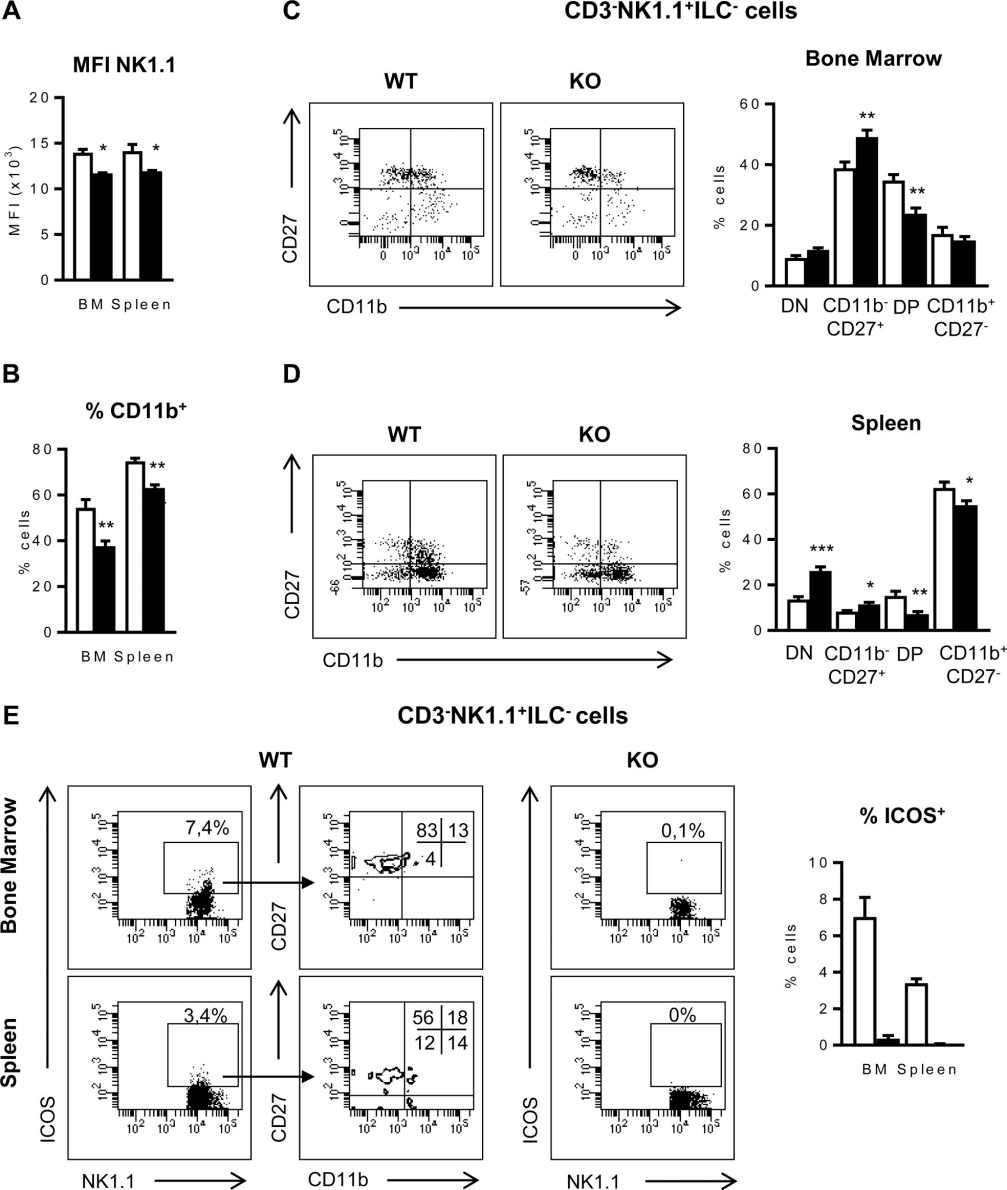
CD27 and CD11b define changes in the maturation of ICOS-KO NK cells. Median of fluorescence intensity (MFI) of NK1.1 (A) and relative number of CD11b^+^ cells (B) in WT and ICOS-KO CD3^-^NK1.1^+^ILC^-^ NK cells (see Methods), from BM and spleen. (C and D) Representative dot-plots and proportion of the different maturation stages based on the expression of CD27 and CD11 in BM (C) and spleen (D) CD3^-^NK1.1^+^ILC^-^ NK cells. (E) Representative dot-plots of ICOS expression in BM and spleen CD3^-^NK1.1^+^ILC^-^ NK cells, and in the different maturation stages. Percentage of ICOS^+^ NK cells is shown at the right. (A-E) Representative dot-plots and mean±SEM of six individual mice per group are shown. Bars, WT (white) and ICOS-KO (black). *p<0.05, **p<0.01, ***p<0.001 between adjacent bars.

As the BM is the main site for NK cell development [49], the fewer NK cells found in the ICOS-KO BM suggests an alteration linked to ICOS deficiency. Thus, NK cells (identified as CD3^-^NK1.1^+^ILC^-^) were further investigated for the acquisition of surface markers like NK1.1, CD11b or CD27 that identify maturation stages in NK cell development [31, 50]. Besides the diminished numbers of CD3^-^NK1.1^+^ILC^-^ cells (Fig 1), membrane NK1.1 expression (Fig 2A) and the presence of CD11b^+^ NK cells (Fig 2B) were reduced in the BM and the spleen of ICOS-KO mice. Then, NK cell maturation stages defined by expression of CD11b and CD27 were analyzed (Fig 2C and 2D), where double negative CD11b^-^CD27^-^NK1.1^+^ cells (DN) are an early stage of NK maturation, followed by the CD11b^-^CD27^+^, double positive CD11b^+^CD27^+^ (DP) and CD11b^+^CD27^-^ stages (most mature) [31]. The frequency of mature NK cells was higher in the spleen than in the BM, and there was an apparent delay of NK maturation in both organs of ICOS-KO mice (Fig 2C and 2D). Fig 2C shows that in the BM, ICOS-KO mice contain a higher proportion of CD11b^-^CD27^+^ NK cells and a lower proportion of DP cells. Similarly, the spleen of these mice contains a higher proportion of immature DN and a lower proportion of DP and CD11b^+^CD27^-^ mature NK cells (Fig 2D), suggesting delayed NK maturation.

ICOS is expressed weakly in resting NK cells in the spleen of WT C57BL/6 mice, yet it is upregulated upon their activation (S1C and S1D Fig, and [6]). However, since there are no data about ICOS levels during NK cell development, stage-specific expression of ICOS was characterized. Fig 2E shows that, in the BM (top panels) or in the spleen (bottom panels), most ICOS^+^CD3^-^NK1.1^+^ILC^-^ NK cells are in the CD11b^-^CD27^+^ compartment, although in the spleen, some ICOS^+^ NKs are also found in the CD11b^+^ compartment.

**Fig 3:**
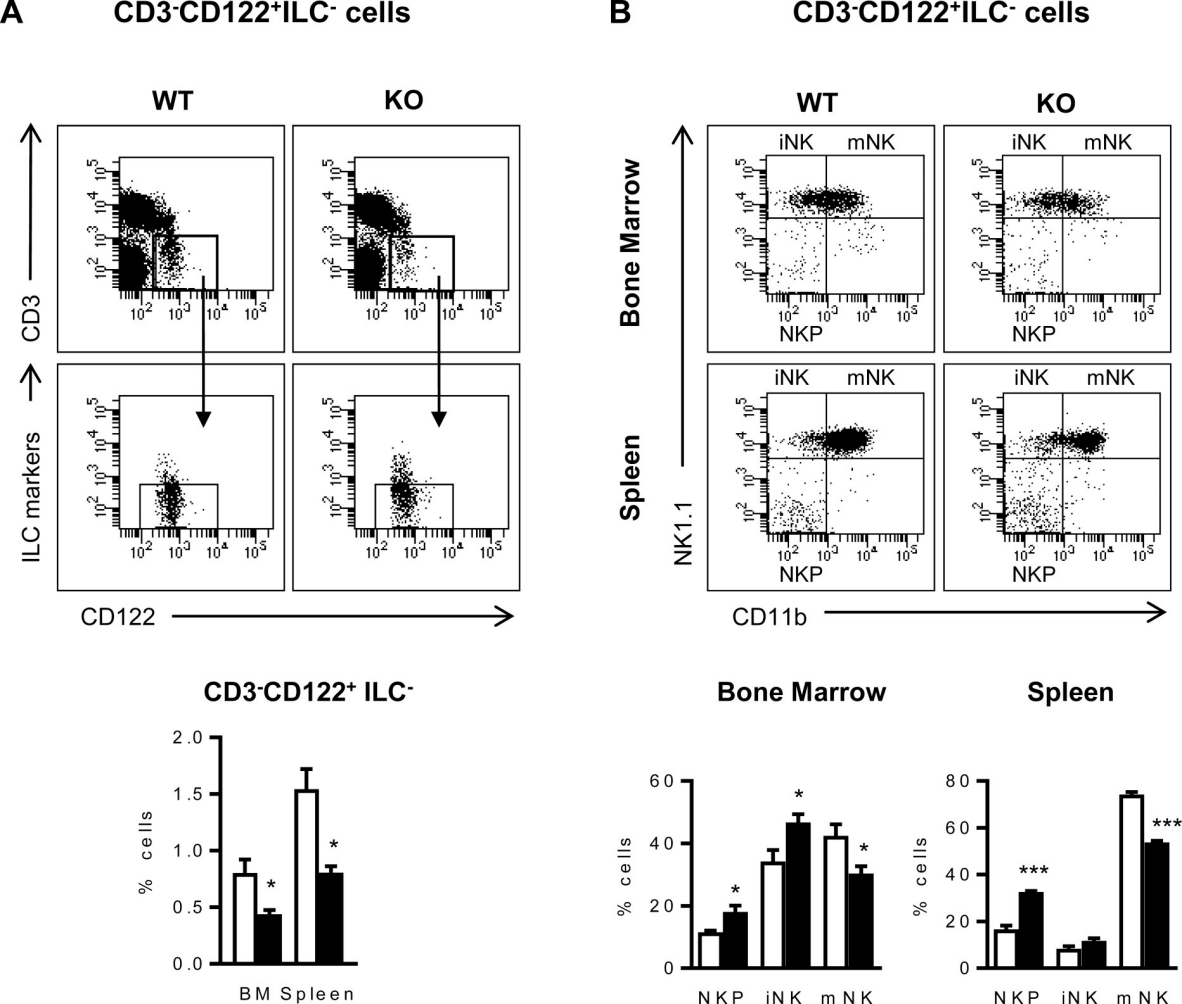
CD3^-^CD122^+^ILC^-^ NK cell maturation stages are altered in ICOS-KO mice. (A) Top, representative dot-plots showing the strategy to select spleen CD3^-^CD122^+^ILC^-^ NK cell populations. Bottom, relative number of CD3^-^CD122^+^ILC^-^ NK cells in the BM and spleen of WT (white bars) and ICOS-KO (black bars) mice. (B) Top, representative dot-plots of NK1.1 and CD11b expression in BM and spleen CD3^-^CD122^+^ILC^-^ NK cells from WT and ICOS-KO mice showing the different maturation stages: NK1.1^-^CD11b^-^ NK cell precursors (NKP), NK1.1^+^CD11b^-^ immature NK cells (iNK) and NK1.1^+^CD11b^+^ mature NK (mNK) cells as indicated. Bottom, relative numbers of the different NK maturation stages based on NK1.1 and CD11b expression in CD3^-^CD122^+^ ILC^-^ cells from the BM or spleen of WT or ICOS-KO mice. (A and B) Histograms show data (mean±SEM) of one representative experiment out of three, each with three individually analyzed mice per group. Bars, WT (white) and ICOS-KO (black). *p<0.05, **p<0.01, ***p<0.001 between adjacent bars.

During the NK cell maturation process, NK cell precursors (NKPs) start to express CD122 but they do not express NK1.1 yet [25, 28], resulting excluded in the previous analysis (Fig 2). Thus, to further study the influence of ICOS deficiency in early stages of NK maturation, the analysis of CD122 expression in NK cell subpopulations was included (Fig 3A). We found significantly fewer CD122^+^CD3^-^ILC^-^ cells in the BM and spleen of ICOS-KO than in WT mice using this type of analysis (Fig 3A, bottom panel). Moreover, CD122^+^CD3^-^ILC^-^ BM and spleen cells from ICOS-KO mice had more double negative NK1.1^-^CD11b^-^ NK cell precursors (NKP) and fewer mature NK1.1^+^CD11b^+^ (mNK) cells than WT counterparts (Fig 3B). An increased proportion of immature iNK cells was also found in the BM of ICOS-KO mice (Fig 3B). Taken together, our results show delayed NK maturation in ICOS-deficient mice.

Hence, ICOS expression may be important to NK cell differentiation in the BM, and ICOS deficiency might be involved in the lower expression of NK1.1 in cells from ICOS-KO mice (Fig 2A), delaying the upregulation of NK1.1 expression during development, the accumulation of the early stage NKP cells and the imbalance of NK cell maturation stages observed in ICOS-KO mice.

### Mouse NK cells and BMDCs can co-express ICOS and its ligand ICOS-L

Signaling through ICOS depends on the interaction with its ligand ICOS-L, which becomes down-modulated upon binding to ICOS [10, 51]. In this sense, higher levels of ICOS-L^+^ from B cells and DCs were detected in ICOS-KO mice (S2A–S2C Fig).

**Fig 4:**
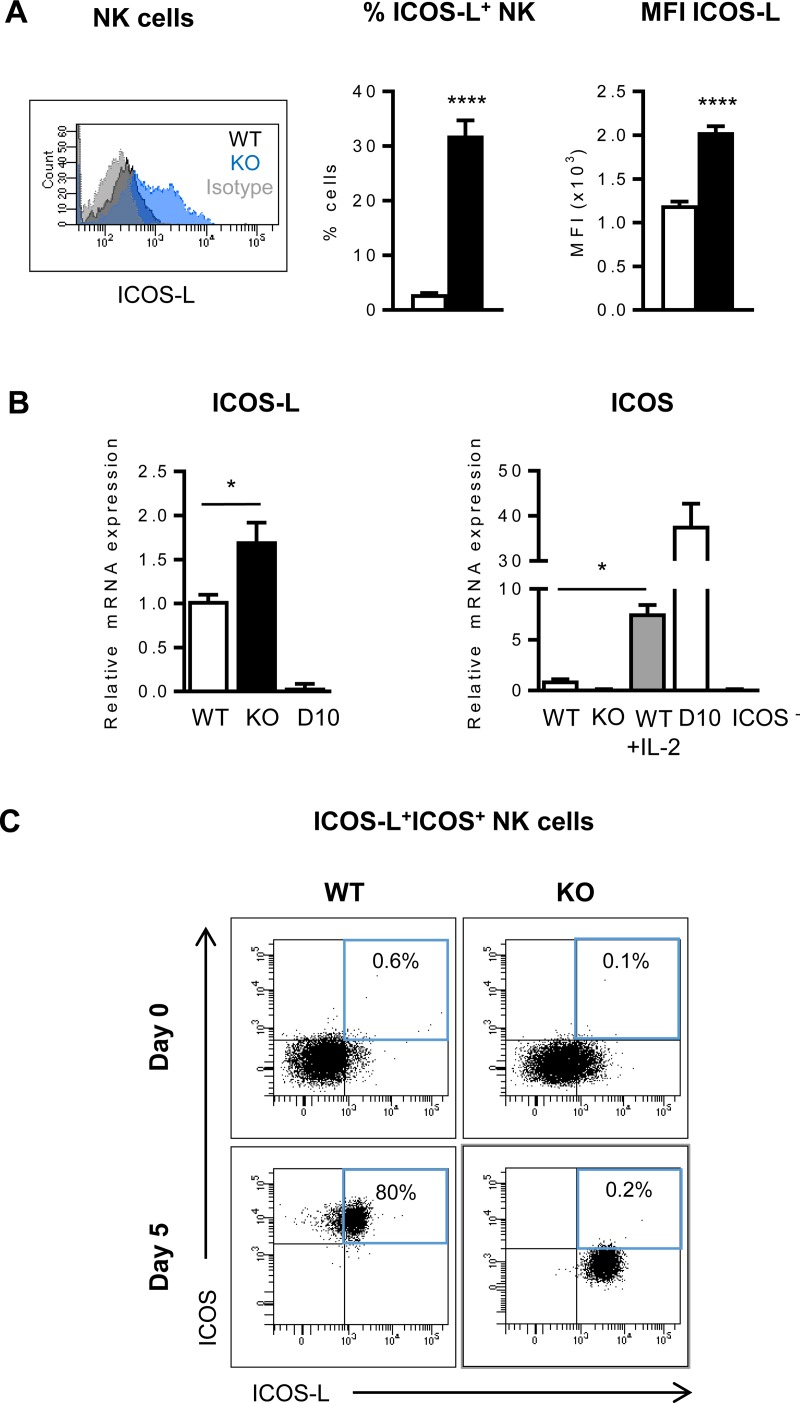
Analysis of ICOS and ICOS-L expression in NK cells. (A) Left, A representative histogram of ICOS-L expression in splenic WT (black) and ICOS-KO (blue) NK cells. Staining with isotype antibody control depicted in gray. Middle and right, relative numbers of ICOS-L^+^ NK cells and their ICOS-L median of fluorescence intensity (MFI) in the spleen of WT (white bars) or ICOS-KO (black bars) mice. Mean±SEM from four independent biological samples are shown. (B) Left, Relative ICOS-L m-RNA expression determined by RT-qPCR in sorted CD3^-^NK1.1^+^ spleen cells from WT (white bars) and ICOS-KO (black bars) mice; SR.D10 T cells (D10) were used as a negative control. Right, ICOS m-RNA expression determined by RT-qPCR in sorted fresh or 5-day IL-2-cultured CD3^-^NK1.1^+^ spleen cells from WT or ICOS-KO mice, as indicated in the graph. SR.D10 or an ICOS^-^ mutant of this cell line were used as positive and negative controls, respectively. Mean±SEM of three independent biological samples analyzed and normalized as described in Methods are shown. *p<0.05, ****p<0.0001 between the indicated bars. (C) Representative dot-plots from one experiment out of three showing ICOS and ICOS-L expression in column-purified NK cells from WT and ICOS-KO mice, before and after culture for 5-days with IL-2. The percentage of co-expression is indicated in the dot-plots.

Intriguingly, we observed that a fraction of BM and spleen NK cells also expressed surface ICOS-L, a phenomenon that was most apparent in ICOS-KO mice (Fig 4A) and it was confirmed at the RNA level (Fig 4B, left). In contrast, ICOS-L was undetectable in the Th2 cell line SR.D10 used as a control [39] (Fig 4B, left). Then, we analyzed whether ICOS and ICOS-L were expressed by the same or different subpopulations of NK cells. Culture of NK cells for five days in the presence of IL-2 induced strong ICOS expression, as confirmed by RT-qPCR (Fig 4B, right) and cytometry (Fig 4C). Interestingly, most IL-2-stimulated NK cells from WT mice co-expressed both, the ICOS costimulatory molecule and its ligand (Fig 4C), suggesting that regulation of ICOS and the ICOS ligand expression differs in NK cells when compared to T or B lymphocytes.

**Fig 5:**
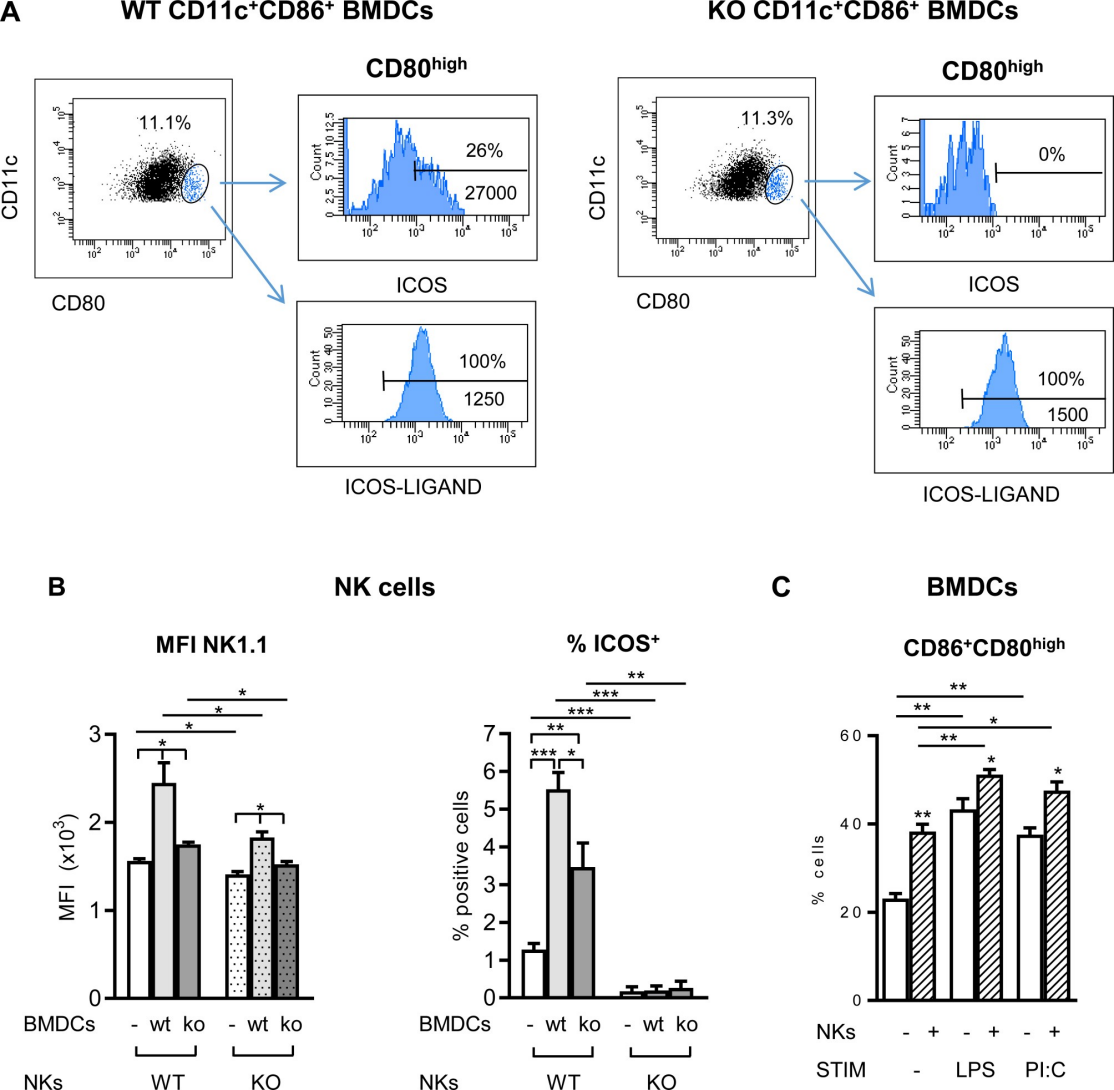
ICOS expressed on NK cells and immature BMDCs modulates cross-talk and cell activation. (A) ICOS and ICOS-L co-expression in CD11c^+^CD86^+^CD80^high^ BMDCs. Histograms showing ICOS and ICOS-Ligand expression in the CD11c^+^CD86^+^CD80^high^ subpopulation (gated in blue in the dot-plots) from WT (left) or ICOS-KO (KO, right) BMDCs. (B) Effect of ICOS expression in NK:BMDCs co-culture. Freshly isolated NK cells from WT (solid bars) or ICOS-KO (dotted bars) mice were cultured for 24 h with WT or ICOS-KO immature BMDCs as indicated in the figure. Expression of NK1.1 (median of fluorescence intensity, MFI) and relative number of ICOS^+^ in the NK cells are shown. (C) BMDCs respond to NK in co-culture. Co-expression of CD80/CD86 in WT BMDCs in the presence (stripped bars) or absence (white bars) of WT NK cells, together with the stimuli indicated (LPS or polyI:C, PI:C). Data are mean±SEM of one representative out of two experiments with three to four biological replicates. *p<0.05, **p<0.01, ***p<0.001 between the indicated bars.

Co-expression of ICOS and ICOS-L has been observed in other innate immune cells including human monocyte-derived dendritic cells [22], and this might be important to NK:DC interactions. We observed that a fraction of WT mouse immature BMDCs gated for CD80^high^ expression co-expressed both ICOS and ICOS-L (Fig 5A), whereas ICOS-L was expressed by all CD11c^+^ BMDCs in both WT and ICOS-KO cells. Co-expression of ICOS and ICOS-L mRNA was confirmed by RT-qPCR in sorted total CD11c^+^ or CD11c^+^CD86^+^CD80^high^ cells (S2D and S2E Fig).

### ICOS/ICOS-L interactions in the homeostasis and activation of NK cells

The co-expression of ICOS and ICOS-L in murine BMDCs and activated NK cells could allow both receptor and ligand signals to these cells, as ICOS reverse signaling through ICOS-L has been described in DCs, fine-tuning the immune response [52, 53]. To analyze the role of ICOS in NK and BMDC interactions, freshly-obtained purified NK cells and immature BMDCs from WT or ICOS-KO mice were co-cultured *in vitro* for 24 h (Fig 5B). Co-culture of WT NK cells with WT immature BMDCs enhanced the expression of functional molecules in NK cells including NK1.1 and ICOS (Fig 5B). Interestingly, the ICOS phenotype of the BMDCs also influenced NK responses. Thus, deficient up-regulation of NK1.1 and ICOS expression was observed in WT NK cells when co-cultured with ICOS-deficient BMDCs (Fig 5B). ICOS-KO NK cells co-cultured with WT immature BMDCs had a weaker response than WT NK cells, as measured by NK1.1 expression level (Fig 5B). ICOS deficiency in both NK and BMDC deeper affected NK1.1 expression in the NK cells (Fig 5B). Interaction of BMDCs with NKs also activated the WT dendritic cells, with enhanced expression of CD80/CD86 (Fig 5C), which was further increased by addition of LPS or poly(I:C) to the co-culture (Fig 5C).

**Fig 6:**
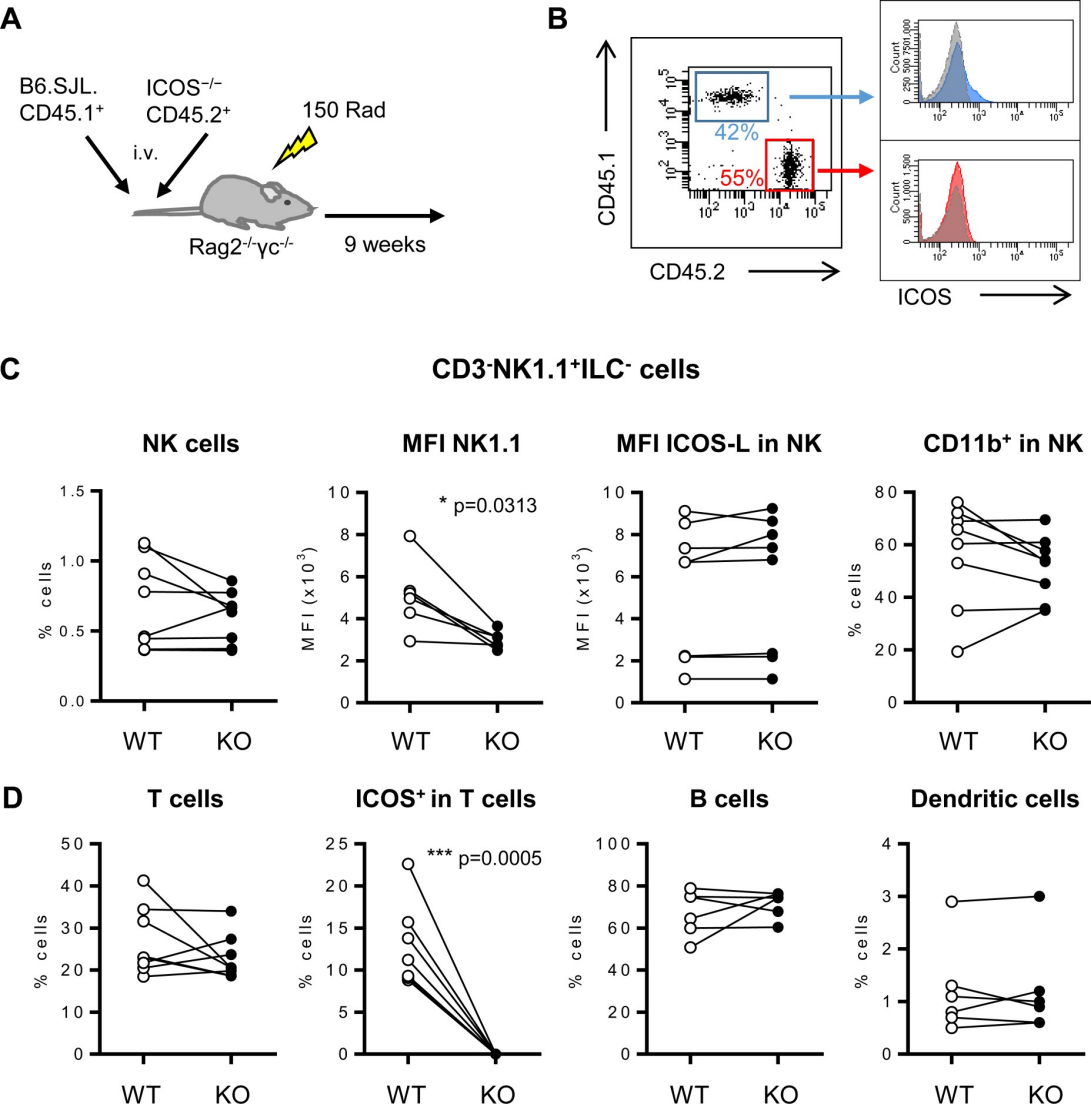
Analysis of mixed bone marrow chimeric mice. Analysis of spleen cells of Rag2^-/-^γc^-/-^recipient mice reconstituted with 10^7^ donor BM cells containing a 1:1 mixture of B6.SJL.CD45.1^+^ (WT CD45.1^+^) plus ICOS^−/−^CD45.2^+^ BM cells. (A) The scheme depicts the mixed BM mouse model generated. (B) Representative dot-plot of the spleen lymphoid cells from one mixed BM chimeric mouse nine weeks after being transferred. ICOS staining for the CD45.1^+^ (blue) and CD45.2^+^ (red) populations is shown at the right; isotype staining control in grey. (C) Analysis of the frequency of positive cells or median of fluorescence intensity (MFI) of the markers NK1.1, ICOS-L and CD11b in the spleen NK cells of mixed BM chimeric mice. NK cells were selected as CD3^-^NK1.1^+^ILC. (D) Analysis of the relative number of T (CD3^+^NK1.1^-^), B (CD19^+^) and DCs (CD11c^high^) cells, and ICOS expression in T cells in the spleen of mixed BM chimeric mice. B6.SJL.CD45.1^+^ (denoted as WT, white circles) and ICOS-KO CD45.2^+^ (KO, black circles). Data (mean±SEM) from two reconstitution experiments, each with four animals, are shown. The eight chimeric mice were analyzed individually. Wilcoxon–matched rank test was used to compare paired results of CD45.1 vs. CD45.2 cells in each mouse. Significant p value as indicated in the graphs.

However, these experimental *in vitro* systems do not distinguish between an intrinsic defect of ICOS-KO NK cells versus an indirect effect due to a deficient environment derived from ICOS deficiency in other cell populations in the mouse. To check these two possibilities, irradiated Rag2^-/-^γc^-/-^recipient mice were i.v. injected a 1:1 mixture of B6.SJL.CD45.1^+^ (WT CD45.1^+^) and ICOS-KO CD45.2^+^ BM cells, and reconstitution was allowed for nine weeks (Fig 6A and 6B). When the spleen of the reconstituted mixed BM chimeras was analyzed and WT CD45.1^+^ versus ICOS-KO CD45.2^+^ populations were compared, the relative numbers of NK (CD3^-^NK1.1^+^ILC^-^) cells from each population did not show significant differences (Fig 6C, left). In contrast, NK1.1 fluorescence intensity in WT CD45.1^+^ was higher than in ICOS-KO CD45.2^+^ NKs (Fig 6C). These results indicate that the defect in NK1.1 expression is a cell-intrinsic characteristic in ICOS-KO NK cells. Hence, ICOS deficiency in NK cells is involved in the lower expression of NK1.1.

ICOS-L expression is higher in cells from ICOS-KO mice (Fig 4 and S2 Fig). However, in the mixed BM chimeric mice, ICOS-L expression was not different in ICOS-KO CD45.2^+^ or WT CD45.1^+^ NK cells (Fig 6C). Thus, ICOS/ICOS-L interaction in the CD45.1^+^ plus CD45.2^+^ mixed BM had been operative, and ICOS-L expression seems to be regulated by the interaction with ICOS expressed in CD45.1 cells.

Other parameters analyzed in the NK cells of the mixed BM chimeras, including the relative numbers of CD11b^+^ cells (Fig 6C), did not show significant differences between CD45.1^+^ and CD45.2^+^ NK cells, indicating that in the chimeric mice the deficient phenotype of ICOS-KO NK cells observed previously had been overcome. Except for the ICOS expression in T cells, no further significant differences in T, B or CD11c^+^ populations were detected between CD45.1^+^ and CD45.2^+^ cells (Fig 6D).

### ICOS- and ICOS-L-mediated signaling in murine NK cells

**Fig 7:**
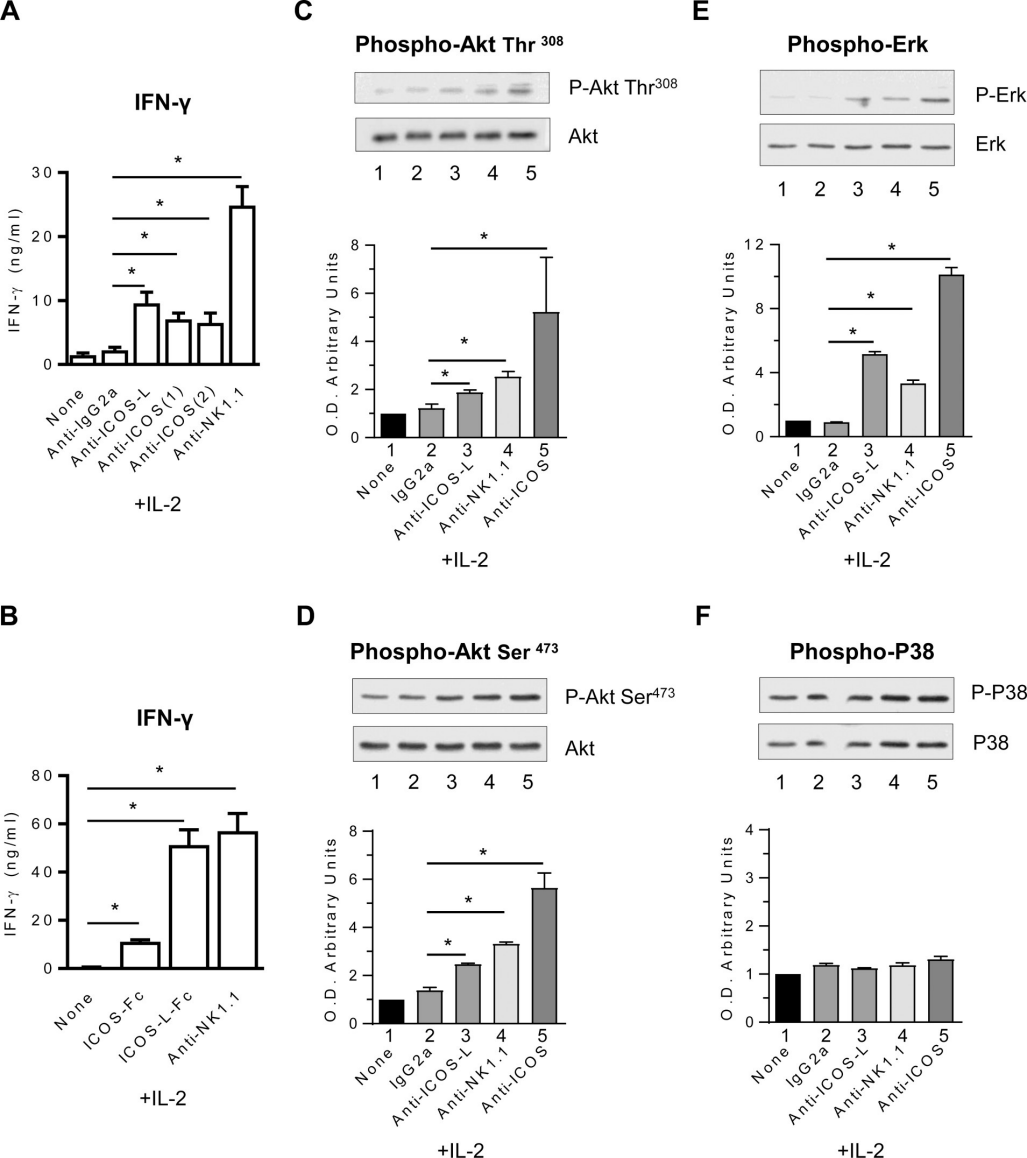
ICOS and ICOS-L costimulate cell signaling and IFN-γ production in NK cells. IFN-γ content in culture supernatants of IL-2-expanded WT NK cells stimulated for 24 h with IL-2 in the presence of plate-adsorbed mAbs (A) or recombinant molecules (B) as indicated. Data (mean±SEM) of two independent experiments with three biological samples each. (C-F) Immunoblot analysis of ICOS and ICOS-L-mediated cell signaling in purified NK cells that were activated for 5 days with IL-2, starved and then eventually stimulated for 30 min with mAbs adsorbed to latex-beads, in the presence of IL-2. Immunoblots probed for phospho-Akt (C and D), phospho-Erk (E), phospho-P38 (F), and their respective loading controls are shown. O.D. ratios were normalized to the value of isotype control (IgG2a, O.D. value = 1). Representative data of three experiments are shown. *p<0.05 between the indicated bars.

While ICOS expression in activated murine NK cells had been described, the expression of ICOS-L by NK cells and its functional role was not known. When NK cells were cultured in the presence of IL-2, the expression of ICOS, ICOS-L, CD28 and CD69 was clearly enhanced (S1D Fig). Simultaneous expression of ICOS and its ligand in IL-2-activated NK cells allowed us to study their signaling potential by ligands including anti-ICOS or anti-ICOS-L mAbs adsorbed to the culture plates. IFN-γ secretion in the culture supernatants after 24 h indicated that two different anti-ICOS mAbs (recognizing two different epitopes), or anti-ICOS-L mAb significantly costimulated IL-2-induced IFN-γ secretion (Fig 7A). Similarly, when fusion proteins of ICOS or ICOS-L and Ig were used as ligands for ICOS-L or ICOS, there was a significant costimulation of IFN-γ secretion by IL-2-activated NK cells (Fig 7B). These results show the capacity not only of ICOS but also of ICOS-L to costimulate NKs and extend to these cells the reverse signaling through ICOS-L previously described in DCs [52, 53]. Hence, signals from both the costimulatory molecule and its ligand influence the activity of NK cells, and the ICOS/ICOS-L interaction appears to have a cell-intrinsic role in the effector response of these cells.

IL-2-expanded NK cells were also used to study early intracellular signaling mediated by surface costimulatory molecules. We used short term (30 min) activation in the presence of IL-2 plus anti-ICOS or anti-ICOS-L mAbs coupled to latex beads, and analyzed Thr^308^- and Ser^473^-Akt-phosphorylation as a measure of PI3K activation as well as phosphorylation of the MAP kinases Erk and P-38. ICOS- and ICOS-L-mediated costimulation increased Akt and Erk phosphorylation (Fig 7C–7E), whereas P-38 phosphorylation was unchanged (Fig 7F). The enhanced early intracellular signaling by both ICOS and ICOS-L, together with the increased IFN-γ secretion, indicates the relevance of these elements in the biology of NK cells.

### ICOS-KO mice show a defective NK response against viral stimulus

To reveal the role of ICOS/ICOS-L interaction in the NK cell response to microbial stimuli, we firstly analyzed the *in vitro* response of WT and ICOS-KO NK cells in the presence of immature BMDCs plus poly(I:C). Our results indicated that impaired ICOS/ICOS-L interactions compromise poly(I:C)-dependent in vitro NK cell activation (S3A Fig) and they predict a deficient *in vivo* NK cell response to poly(I:C) which was corroborated in the ICOS-KO mice (S3B and S3C Fig). They also suggested an implication of those interactions in NK responses to microbial stimuli *in vivo*, as ICOS-KO NK cells respond poorly to poly(I:C) which is a TLR agonist associated with viral infection.

**Fig 8:**
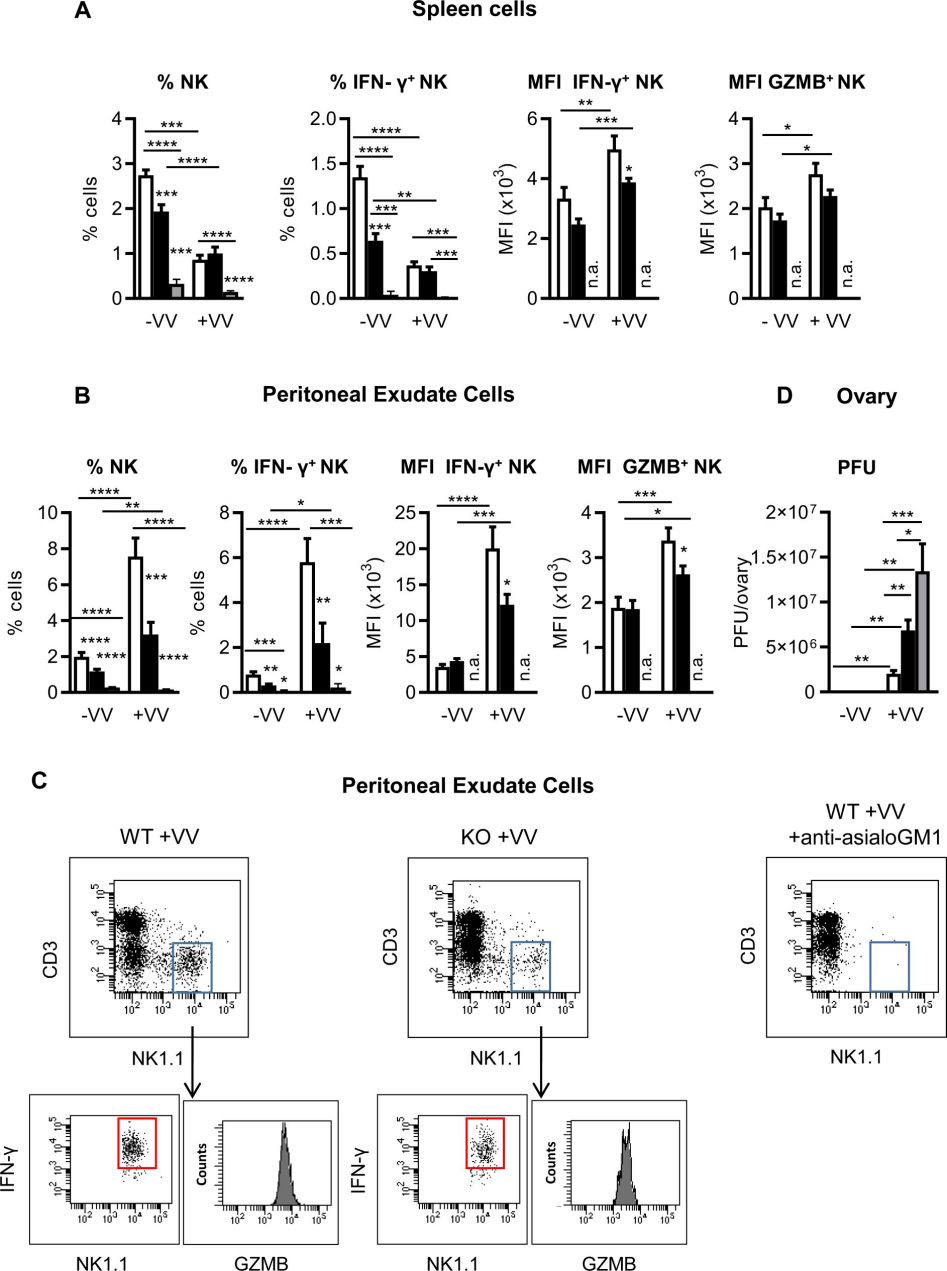
ICOS-KO mice show a defective NK cell-dependent *in vivo* response against Vaccinia virus. Analysis of the *in vivo* NK cell response to vaccinia virus in WT, ICOS-KO mice or NK-depleted WT mice by i.v. injection of anti-asialo-GM1 Ab (as described in Methods section). Mice were infected with 10^6^ pfu of vaccinia virus (+VV) or left uninfected (-VV) and sacrificed 48 h later. Analysis of the relative number of total or IFN-γ^+^NK cells in the spleen (A) or peritoneal exudate (B) of WT (white bars), ICOS-KO (black bars) or NK-depleted WT (gray bars) mice is shown. Median of fluorescence intensity (MFI) of IFN-γ^+^ or granzyme B^+^ in NK cells is also depicted (A-B). (C) Flow-plots of a representative experiment showing the IFN-γ^+^NK subpopulation in the PEC of vaccinia-infected WT and ICOS-KO mice. Granzyme B expression in NK population is shown in the histograms. NK-depletion in the peritoneal exudate of a representative anti-asialo-GM1-treated infected mouse is also shown (C, right). (D) Viral load determined as the viral titer in homogenized ovarian tissue from WT (white), ICOS-KO (black) or NK-depleted (gray) mice. (A-C) Data are mean ±SEM of three infection experiments with vaccinia virus, with N = 9 mice for WT and ICOS-KO groups and N = 6 mice for anti-asialo-GM1-treated mice. The animals were individually processed and analyzed. *p<0.05, **p<0.01, ***p<0.001, ****p<0.0001 between adjacent or the indicated bars. n.a., not applicable.

To study the NK cell response in a more physiological context we aimed to analyze a NK-mediated anti-viral immune response in vaccinia virus infected ICOS-KO versus WT mice. To better check the implication of NK cells in the *in vivo* response, another experimental group was set up in which NK cells were depleted by *in vivo* treatment with anti-asialo-GM1 Ab, as described in the Methods section. Female mice were infected i.p. with vaccinia virus and analyzed 48 h later, before the primary adaptive effector immune response could be effective. Their spleen and peritoneal exudate cells were studied for NK content and function (Fig 8A–8C); and viral load was determined in their ovaries (Fig 8D). Under basal conditions, the NK cell fraction was smaller in the spleen and peritoneal fluid of ICOS-KO mice than in WT mice and was strongly depleted in anti-asialo-GM1 treated mice (Fig 8A and 8B, left). Upon infection there were fewer NK cells in the spleen (Fig 8A left panel), but more in the site of inoculation, the peritoneum (Fig 8B left panel) of both WT and ICOS-KO mice. In addition, the NK cell fraction in the PEC of infected ICOS-KO was significantly lower than in WT mice (Fig 8B, left). The spleen and PEC of anti-asialo-GM1 treated mice group remained NK-depleted upon vaccinia virus infection.

The functional capacity of ICOS-KO NK cells was lower, as measured by the relative number of IFN-γ-producing NK cells in the spleen and peritoneal fluid of ICOS-KO mice in basal conditions (Fig 8A and 8B). After i.p. virus infection, the fraction of IFN-γ^+^ NK cells in the PEC was enhanced in both ICOS-KO and WT mice (Fig 8B). Yet, the response to vaccinia virus in the spleen and PEC of infected ICOS-KO mice was suboptimal in terms of IFN-γ production by NK cells (Fig 8A and 8B, MIF IFNγ^+^ NK). Viral infection also increased the expression of granzyme B^+^ in the spleen and peritoneal exudate NK cells (Fig 8A and 8B); but this response was lower in the NK PEC of ICOS-KO mice (Fig 8B).

The efficacy of virus clearance was determined by titrating the virus in the ovary of infected mice, a site of viral accumulation (Fig 8D). The results obtained at 48 h, before the primary adaptive immune response is effective, reflect the NK cell response. Indeed, the viral load in the ovary of infected animals is strongly augmented in anti-asialo-GM1-treated as compared to WT mice, indicating a deficient viral clearance and *in vivo* response in the case of NK cell depletion (Fig 8D).Virus content in the ovary of ICOS-KO mice represents an intermediate situation between WT and NK-depleted mice. Although NK cells remain present in the infected ICOS-KO mice, functional defects are patent (Fig 8A–8C) which may justity a deficient *in vivo* anti-viral response. These results show the importance of ICOS-mediated signaling for NK cell activation and the control of viral infection *in vivo*.

## Discussion

ICOS deficiency causes common-variable-immunodeficiency in humans [12] which has been recently reclassified as a combined immunodeficiency [54]. A follow-up of the patients has shown increased susceptibility to viral and opportunistic infections, as well as cancer [19]; this suggests that innate immune responses might be also affected. However, the implication of the ICOS/ICOS-L axis in the biology of innate natural killer cells has been barely explored. Using ICOS-deficient mice, we show here a role of ICOS in the homeostasis, development and function of NK cells with consequences to NK-dependent responses against *in vivo* viral infection. Our data showing that ICOS-deficient mice have fewer NK cells in their BM and spleen, which also show higher rates of spontaneous apoptosis, suggest a formerly unknown implication of ICOS in NK cell homeostasis and survival. These findings might explain very recent reports on ICOS or ICOS-L human immunodeficiency in which NK cells lymphopenia is often found [19, 55].

Here we also demonstrate a delay in the maturation stages of ICOS-KO NKs in the spleen and in the BM, which is the primary organ for NK cell development. Thus, cells accumulate at the immature stage CD27^+^CD11b^-^CD3^-^NK1.1^+^CD49a^-^CD49d^-^IL33R^-^RORγT^-^, actually a stage in which we have detected expression of ICOS in NKs. Alteration in maturation stages can trigger functional defects in the whole population of NK cells, as different stages may have different functional capabilities [29] and account for NK cell dysfunction [56]. Besides the defects in NK homeostasis and maturation, we have also observed functional defects in ICOS-KO NK cells. So, the cytotoxic activity of IL-2-expanded NKs from ICOS-KO mice is decreased against tumor cells; which is remarkable when target cells expressing high levels of ICOS-L are used (S4 Fig). This is in agreement with previous data showing that blocking antibodies impaired ICOS costimulation of cytotoxic activity and IFN-γ production in activated murine NK cells [6]. In addition, *in vitro* and *in vivo* experiments analyzing the response of NK cells to poly(I:C) show functional defects in ICOS-KO NK with impaired expression of NK1.1 and CD69 (S3 Fig). The lower IFN-γ levels found in the sera of ICOS-KO mice treated with poly(I:C) (S3 Fig) extend and confirm the data on surface markers and reflect a weaker response of ICOS-KO NK cells.

Whereas the role of ICOS in infection is well established in T cells [57, 58] data on NK cell-mediated responses are lacking. Our studies show significant defects in the response of ICOS-KO NK cells to vaccinia virus *in vivo*, as shown by the percentage of total NK, IFN-γ-secreting NK cells, and the expression of granzyme B^+^ in the spleen or the peritoneal exudate of infected mice, together with higher vaccinia virus titers in the ovaries of ICOS-KO infected mice. The functional defects that we show in ICOS-KO mice are supported by data demonstrating that impaired ICOS costimulation decreases cytotoxic activity in activated murine NK cells (S4 Fig and [6]). Our findings are also in agreement with the decreased NK cell counts and the increased susceptibility to viral and opportunistic infection, as well as cancer recently described in ICOS- or ICOS-L-deficient humans [19, 55].

Another remarkable finding of our data is the co-expression of ICOS and its ligand ICOS-L by mouse BMDCs and IL-2-activated-NK cells, with signaling capacity in NK cells. ICOS and ICOS-L are expressed separately in adaptive immune cells like T or B lymphocytes. Moreover, surface ICOS-L in B cells and APCs is down-modulated mainly by ICOS^+^ T lymphocytes [10]. ICOS and ICOS-L co-expression has been described in other innate immune cells, such as murine ILC2 cells [5, 59] and human BMDCs [22]. The reason for this permissive co-expression of the costimulatory molecule and its ligand in innate cells is presently unknown, but it makes homotypic (or *cis*) as well as heterotypic (or *trans*) interactions feasible with interesting connotations at the biological level. In ILC2 cells it has been proposed that *cis* homotypic interactions participate in the production of cytokines necessary for homeostasis, like IL-13 [5, 60]. In human BMDCs, homotypic ICOS/ICOS-L interactions costimulate intracellular signals that enhance cytokine production [22]. One could hypothesize a similar effect in resting murine NK cells which show a low ICOS and ICOS-L expression, yet their signals could be sufficient to produce the cytokines needed to maintain homeostasis, and hypothetically explain the deficient NK cell numbers found in ICOS-KO mice reported here or in ICOS/ICOS-L-deficiency in humans.

In the heterotypic interactions, the receptor and its ligand expressed on different cells belonging to the same [60] or different cell populations might broaden the possibilities for NK cells to stablish cell-to-cell interactions. In fact, we show that heterotypic interactions during co-culture of resting NK cells with BMDCs, result in activation of both types of cells. Thus, ICOS-deficiency in either NK or immature BMDC impairs up-regulation of activation markers like NK1.1 or ICOS in the NK cells. These results also suggest a functional link between ICOS and NK1.1 expression as a marker of NK maturation and activation.

The defects in homeostasis and maturation of NK cells in ICOS-KO mice could be due to an intrinsic defect of NK cells (i.e., impaired ICOS- or ICOS-L-mediated signals, see below). Alternatively, an ICOS-dependent effect on other cell populations (i.e., provoking an altered cytokine profile) with an impact on NK cells cannot be ruled out. No defects were observed in the efficiency of reconstitution of irradiated Rag2^-/-^γc^-/-^ mice by ICOS-KO BM cells regarding NK and other cell populations, as compared to WT BM. This suggests that whenever ICOS^+^ cells are present, they may directly or indirectly compensate for ICOS-deficiency in the NK cells, normalizing the homeostasis of this cell population. However, the lower NK1.1 expression levels found in ICOS-KO NK cells were not augmented in the mixed BM chimeric mice, indicating a cell-intrinsic defect in these cells at this level, and also showing a link between ICOS and NK1.1 expression. Thus, ICOS-KO NKs show two types of defects: a cell-intrinsic one regarding the modulation of NK1.1 levels, and others caused by the deficient milieu as a consequence of the lack of ICOS in other cells.

We have shown that ICOS-deficiency provokes a hyper-expression of ICOS-L in various cell populations including ICOS-KO NK cells. Interestingly, the higher ICOS-L levels in ICOS-KO NK cells were decreased to the level found in WT NK in the chimeric mice, indicating that functional interactions with ICOS^+^ WT cells have occurred. This is important, as our data indicate that not only ICOS, but also ICOS-L ligation, enhance early Akt and Erk phosphorylation, or IFN-γ secretion in IL2-activated NK cells. ICOS reverse signaling through ICOS-L has been formerly described in DCs, fine-tuning the immune response [52, 53]. Our results suggest reverse signaling through ICOS-L acting in NK cells and some degree of redundancy in ICOS and ICOS-L intracellular signals. However, fine-tuning of NK cell responses by differential activation of other signaling pathways by ICOS or ICOS-L cannot be ruled out. Basal expression of ICOS-L together with their signaling capacity might hypothetically ensure effective ICOS trans-activation of NK cells in the early phases of the immune response, when ICOS expression by NKs is still low, or during NK cell differentiation. In our mixed BM chimera experiments, ICOS-L expressed by ICOS-KO CD45.2^+^ NK cells could promote intra-cell signaling when interacting with ICOS^+^ CD45.1^+^ cells, and that could favor the development and homeostasis of CD45.2^+^ NK cells harboring ICOS-L.

Thus, our experiments with chimeric mice and cell signaling point aspects of NK cell biology differently affected by ICOS-deficiency, with some of them which may be dependent on ICOS-L signaling or extrinsic to the ICOS-deficiency in the NK cells.

Overall, our results show a role for ICOS and its ligand in the NK cell mediated immune response and in their relationships with other cellular populations. The complexity of these effects is augmented by simultaneous co-expression of ICOS and ICOS-L in innate immune cells, as exemplified by the murine IL-2-stimulated NK cells and BMDCs described here. The signaling capacity of these molecules in NK cells suggest potential avenues for the use of recombinant constructs of ICOS and ICOS-L, or specific Abs for the therapeutic manipulation of NK immune responses and provides the basis for future studies in which the targeting of the ICOS/ICOS-L axis may support new strategies to characterize and manage these immunodeficiencies. Our results also highlight the increased parallelism found between ICOS KO mice and ICOS-immunodeficient patients suggesting the utility of the animal model to study and assay new therapies and modulators of costimulation signaling.

## Supporting information

S1 Fig The response of NK cells to IL-2. (A) Proliferation curves of WT and ICOS-KO purified NK cells, activated with IL-2 (2,000 U/ml) or IL-15 (100 ng/ml) for up to 7 days. Cells were stained with trypan blue and counted in a Neubauer hemocytometer. (B) STAT5 phosphorylation (pSTAT5) in WT (blue histogram) and ICOS-KO (pink) NK cells activated for 5 days with IL-2, starved and activated for 30 min with IL-2. Isotype control in gray; the median of fluorescence intensity (MFI) is shown in the histogram. (C) ICOS expression in magnetic-column purified spleen NK WT fresh cells (gray) or after *in vitro* culture with IL-2 (2,000 U/ml) for 48h (light blue) or 72h (dark blue). The MFI is indicated for each peak. (D) Expression kinetics of activation surface markers (ICOS, ICOS-L, CD28 and CD69) in WT and ICOS-KO NK cells purified from spleen and cultured with 2,000 U/ml IL-2 for the times indicated. (A and D) Mean±SEM from two to three experiments, each with three biological replicates, is shown. (B and C) Histograms showing one representative of three experiments. Symbols: WT (white symbols) ICOS-KO (black symbols).
(PDF)

S2 Fig ICOS-deficiency increases ICOS-L expression at the cell surface. ICOS-L expression in (A) total lymphoid, (B) CD19^+^ or (C) CD11c^high^ bone marrow (BM) and spleen cells. Top, percentage of ICOS-L^+^ cells in each cell type from WT (white) or ICOS-KO (black) mice. ICOS-L median of fluorescence intensity of isotype control staining (pink)/ICOS-L staining in WT (blue)/ICOS-L staining in ICOS-KO (gray) cells are shown in brackets. Data from three biological replicates. *p<0.05 between adjacent bars. Bottom, representative histograms of WT (blue) and ICOS-KO (gray) cells. **ICOS and ICOS-L expression in murine bone marrow-derived dendritic cells.** (D) ICOS mRNA expression determined by RT-qPCR in sorted CD11c^+^ cells WT or ICOS-KO and CD11c^+^CD86^+^CD80^++^ WT (CD11c^+^CD80^++^ WT) cells. SR.D10 and an ICOS-deficient mutant cell line were used as positive and negative controls, respectively. (E) ICOS-L mRNA expression in sorted CD11c^+^ BMDC (WT, KO) was determined by RT-qPCR. SR.D10 cells were used as a negative control. (D) and (E) are data from three independent experiments normalized to the TBP gene and relative to the WT total CD11c^+^ BMDC expression (value 1). *p<0.05 between the indicated bars.
(PDF)

S3 Fig In vitro and in vivo defective ICOS-KO NK cell responses to poly(I:C). **(A)** *In vitro* NK cell responses to Poly(I:C): Fresh, column-purified WT and KO NK cells were co-cultured for 24 h with respectively matched WT or KO BMDCs in the presence or absence of Poly(I:C), at different concentrations (1–10 μg/ml). Modification of NK cell activation markers such as NK1.1, CD69 and ICOS was assessed. Data (mean±SEM) of three to four independent biological samples. *p<0.05, between adjacent bars or as indicated. B) *In vivo* response of WT and ICOS-KO mice injected with poly(I:C) (150 μg in PBS, i.p.). Percentage and number of NK cells, IFN-γ-producing NK cells, and the expression of NK activation markers, including NK1.1 and CD69, in peritoneal exudate cells (PEC). PEC were obtained 18 h post-poly(I:C) injection. (C) IFN-γ levels in the sera of WT and ICOS-KO mice injected with poly(I:C). Sera were obtained 8 h post-poly(I:C)-inoculation. Bars: WT (white), ICOS-KO (black). Data (mean±SEM) of three mice analyzed are shown. *p<0.05, ** p<0.01 between adjacent bars or as indicated. **Materials and Methods: Response of NK cells to poly(I:C) *in vivo*.** Mice were inoculated intraperitoneally (i.p.) with 150 μg of polyinosinic-polycytidylic acid (poly(I:C), Invivo, San Diego, CA, USA) in 0.1 ml of sterile pyrogen-free PBS (Sigma-Aldrich). Control mice received PBS alone under the same conditions. After 8 hours, the animals were anesthetized, bled and their serum was collected for cytokine determination. The mice were sacrificed 18 hours later, peritoneal exudate cell suspension was prepared, and NK cells analyzed by flow cytometry.
(PDF)

S4 Fig ICOS-KO mice show defects in their in vitro NK cytotoxic response. (A) Specific cytotoxicity against a CFSE-labelled YAC-1 tumor cell line of fresh, purified (left) or IL-2 expanded (right) WT (white symbols) or ICOS-KO (black symbols) NK cells, as indicated in the graphs. *p<0.05 between WT and ICOS-KO. (B) ICOS-L expression on target tumor cells enhances the specific cytotoxicity of NK cells. Left, ICOS-L expression in ICOS-L transfected melanoma B16 cells (blue histogram), control transfectants (black) or isotype-control (gray). Right, specific cytotoxicity of IL-2 activated WT or ICOS-KO NK cells against CFSE-labelled ICOS-L transfected B16 melanoma cells: White squares, WT NK cells plus control B16-neo transfectants; white circles, WT NK cells plus B16-ICOS-Lhigh targets; black circles, ICOS-KO NK cells plus B16-ICOS-Lhigh cells. (A) and (B) The effector:target ratio (E:T) is indicated, showing the data from one representative experiment of three analyzed. *p<0.05 comparing NK WT:B16-neo versus NK WT:B16-ICOS-L^high^ NK; § p<0.05 comparing NK WT: B16-ICOS-L^high^ versus NK KO: B16-ICOS-L^high^. **Materials and Methods for Cytotoxicity assay.** Freshly obtained NK cells purified from the spleen, or 6-day IL-2-activated NK cells were used as effectors in cytotoxicity assays against YAC-1 or B16.F10 cell lines. For their use as targets, these tumor cell lines were labeled with CFSE, and cytotoxicity was quantified by flow cytometry ^(1)^. In sterile 96-well round bottom plates, 104 CFSE-labeled target cells/well and a varying number of effector cells (in triplicate) were mixed, giving the indicated effector:target (E:T) ratios. Cultures were incubated for 4 h at 37°C in 5% CO2, and then propidium iodide (PI) was added to the samples and they were incubated for 5 min on ice. After PI staining, the samples were analyzed on a FACSCanto (BD) using the DIVA software (BD). Because cytolysis disrupts cell architecture, the frequency of PI^-^CFSE^+^ live target cells was recorded for each E:T and the relative sample lysis was calculated as 100 − % live cells. The specific cytotoxicity (% CTX) for each sample was calculated as: % CTX = 100 x (% Sample lysis—% Basal lysis) / 100 - % Basal lysis. Basal lysis was calculated as the cell lysis in the absence of effector cells. ^(1)^ Lecoeur, H., et al., A novel flow cytometric assay for quantitation and multiparametric characterization of cell-mediated cytotoxicity. J Immunol Methods, 2001. 253(1–2): p. 177–87.
(PDF)

S1 File Montes-Casado_Original immunoblots. The original western-blots for Fig 7 are shown.
(PDF)
