# Supplementary figures and images for: ICOS deficiency hampers the homeostasis, development and function of NK cells
Source: PLoS One. 2019 Jul 8;14(7):e0219449. doi: 10.1371/journal.pone.0219449 (PMC6613708; doi:10.1371/journal.pone.0219449)

**A**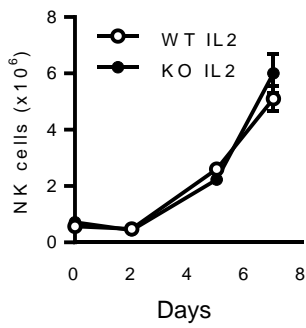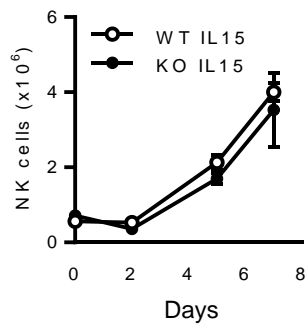**B**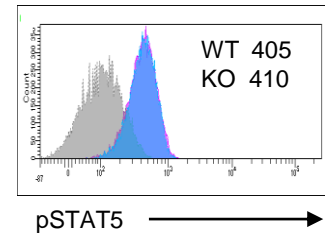**C**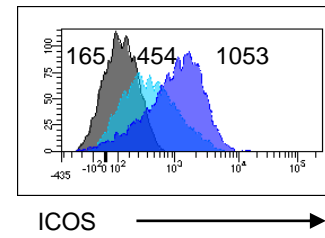**D**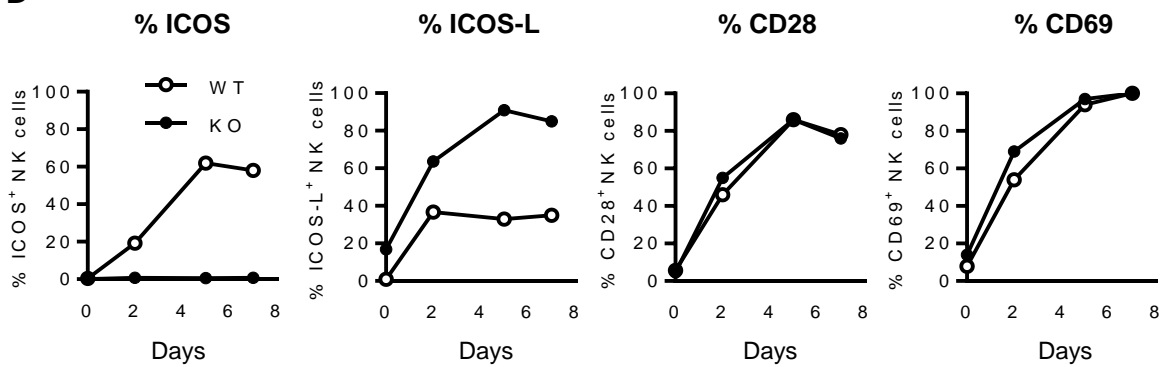**S1 Fig**

Supplement: S1 Fig — (A) Proliferation curves of WT and ICOS-KO purified NK cells, activated with IL-2 (2,000 U/ml) or IL-15 (100 ng/ml) for up to 7 days. Cells were stained with trypan blue and counted in a Neubauer hemocytometer. (B) STAT5 phosphorylation (pSTAT5) in WT (blue histogram) and ICOS-KO (pink) NK cells activated for 5 days with IL-2, starved and activated for 30 min with IL-2. Isotype control in gray; the median of fluorescence intensity (MFI) is shown in the histogram. (C) ICOS expression in magnetic-column purified spleen NK WT fresh cells (gray) or after in vitro culture with IL-2 (2,000 U/ml) for 48h (light blue) or 72h (dark blue). The MFI is indicated for each peak. (D) Expression kinetics of activation surface markers (ICOS, ICOS-L, CD28 and CD69) in WT and ICOS-KO NK cells purified from spleen and cultured with 2,000 U/ml IL-2 for the times indicated. (A and D) Mean±SEM from two to three experiments, each with three biological replicates, is shown. (B and C) Histograms showing one representative of three experiments. Symbols: WT (white symbols) ICOS-KO (black symbols). (PDF) [file pone.0219449.s001.pdf]

### A Lymphoid cells

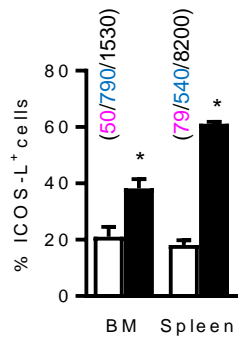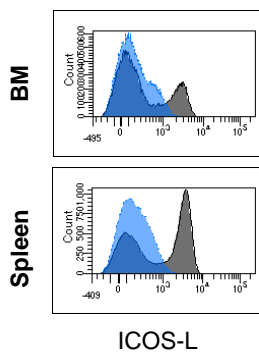

### B CD19+ B cells

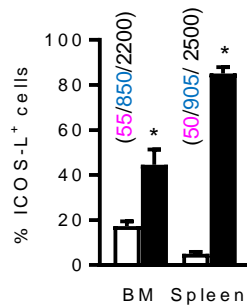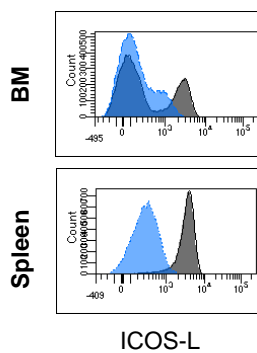

### C CD11c<sup>high</sup> cells

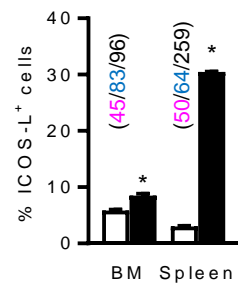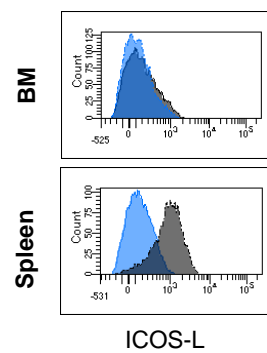

### D ICOS

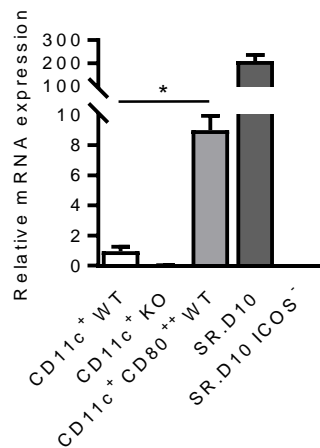

### E ICOS-L

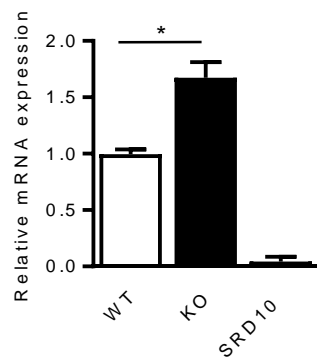

S2 Fig

Supplement: S2 Fig — ICOS-L expression in (A) total lymphoid, (B) CD19+ or (C) CD11chigh bone marrow (BM) and spleen cells. Top, percentage of ICOS-L+ cells in each cell type from WT (white) or ICOS-KO (black) mice. ICOS-L median of fluorescence intensity of isotype control staining (pink)/ICOS-L staining in WT (blue)/ICOS-L staining in ICOS-KO (gray) cells are shown in brackets. Data from three biological replicates. *p<0.05 between adjacent bars. Bottom, representative histograms of WT (blue) and ICOS-KO (gray) cells. ICOS and ICOS-L expression in murine bone marrow-derived dendritic cells. (D) ICOS mRNA expression determined by RT-qPCR in sorted CD11c+ cells WT or ICOS-KO and CD11c+CD86+CD80++ WT (CD11c+CD80++ WT) cells. SR.D10 and an ICOS-deficient mutant cell line were used as positive and negative controls, respectively. (E) ICOS-L mRNA expression in sorted CD11c+ BMDC (WT, KO) was determined by RT-qPCR. SR.D10 cells were used as a negative control. (D) and (E) are data from three independent experiments normalized to the TBP gene and relative to the WT total CD11c+ BMDC expression (value 1). *p<0.05 between the indicated bars. (PDF) [file pone.0219449.s002.pdf]

**A**

***In vitro* NK cell response**

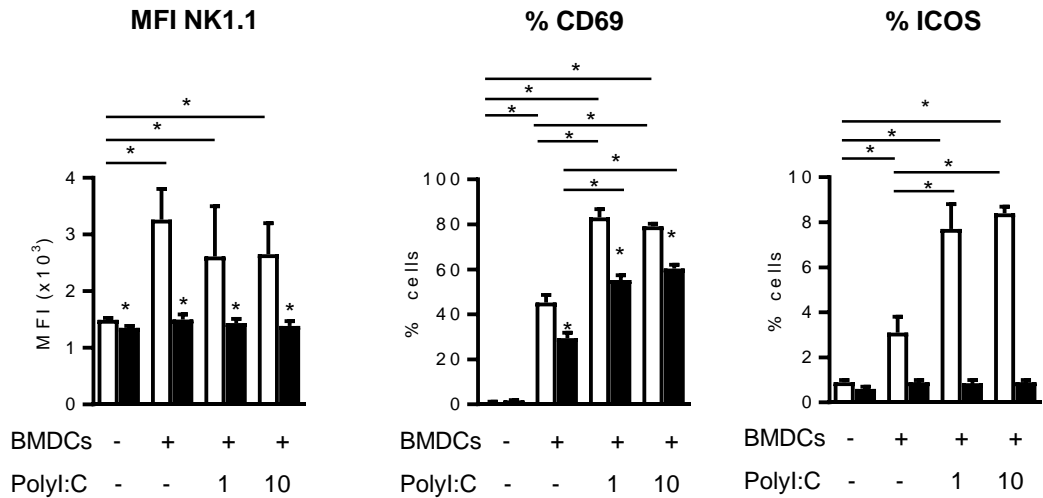

***In vivo* NK cell response**

**B**

**Peritoneal exudate cells**

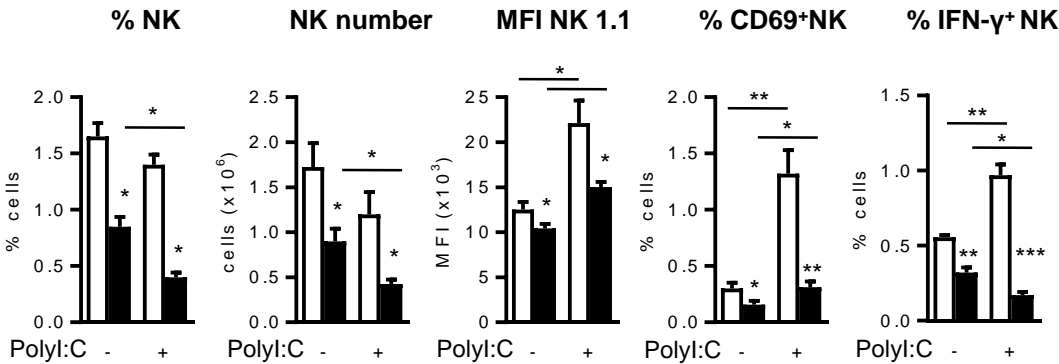

**C**

**Serum**

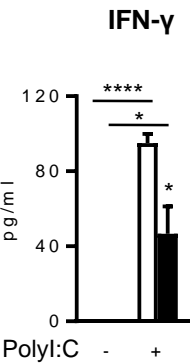

**S3 Fig**

Supplement: S3 Fig — (A) In vitro NK cell responses to Poly(I:C): Fresh, column-purified WT and KO NK cells were co-cultured for 24 h with respectively matched WT or KO BMDCs in the presence or absence of Poly(I:C), at different concentrations (1–10 μg/ml). Modification of NK cell activation markers such as NK1.1, CD69 and ICOS was assessed. Data (mean±SEM) of three to four independent biological samples. *p<0.05, between adjacent bars or as indicated. B) In vivo response of WT and ICOS-KO mice injected with poly(I:C) (150 μg in PBS, i.p.). Percentage and number of NK cells, IFN-γ-producing NK cells, and the expression of NK activation markers, including NK1.1 and CD69, in peritoneal exudate cells (PEC). PEC were obtained 18 h post-poly(I:C) injection. (C) IFN-γ levels in the sera of WT and ICOS-KO mice injected with poly(I:C). Sera were obtained 8 h post-poly(I:C)-inoculation. Bars: WT (white), ICOS-KO (black). Data (mean±SEM) of three mice analyzed are shown. *p<0.05, ** p<0.01 between adjacent bars or as indicated. Materials and Methods: Response of NK cells to poly(I:C) in vivo. Mice were inoculated intraperitoneally (i.p.) with 150 μg of polyinosinic-polycytidylic acid (poly(I:C), Invivo, San Diego, CA, USA) in 0.1 ml of sterile pyrogen-free PBS (Sigma-Aldrich). Control mice received PBS alone under the same conditions. After 8 hours, the animals were anesthetized, bled and their serum was collected for cytokine determination. The mice were sacrificed 18 hours later, peritoneal exudate cell suspension was prepared, and NK cells analyzed by flow cytometry. (PDF) [file pone.0219449.s003.pdf]

**A****YAC-1 cytotoxicity**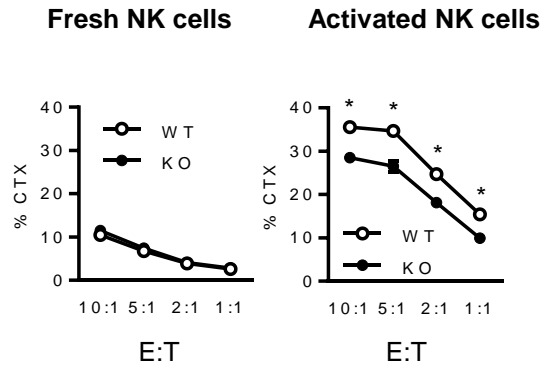**B****B16 cytotoxicity**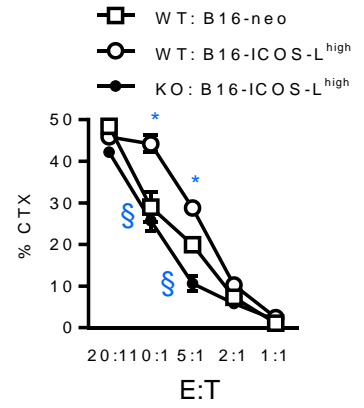**S4 Fig**

Supplement: S4 Fig — (A) Specific cytotoxicity against a CFSE-labelled YAC-1 tumor cell line of fresh, purified (left) or IL-2 expanded (right) WT (white symbols) or ICOS-KO (black symbols) NK cells, as indicated in the graphs. *p<0.05 between WT and ICOS-KO. (B) ICOS-L expression on target tumor cells enhances the specific cytotoxicity of NK cells. Left, ICOS-L expression in ICOS-L transfected melanoma B16 cells (blue histogram), control transfectants (black) or isotype-control (gray). Right, specific cytotoxicity of IL-2 activated WT or ICOS-KO NK cells against CFSE-labelled ICOS-L transfected B16 melanoma cells: White squares, WT NK cells plus control B16-neo transfectants; white circles, WT NK cells plus B16-ICOS-Lhigh targets; black circles, ICOS-KO NK cells plus B16-ICOS-Lhigh cells. (A) and (B) The effector:target ratio (E:T) is indicated, showing the data from one representative experiment of three analyzed. *p<0.05 comparing NK WT:B16-neo versus NK WT:B16-ICOS-Lhigh NK; § p<0.05 comparing NK WT: B16-ICOS-Lhigh versus NK KO: B16-ICOS-Lhigh. Materials and Methods for Cytotoxicity assay. Freshly obtained NK cells purified from the spleen, or 6-day IL-2-activated NK cells were used as effectors in cytotoxicity assays against YAC-1 or B16.F10 cell lines. For their use as targets, these tumor cell lines were labeled with CFSE, and cytotoxicity was quantified by flow cytometry (1). In sterile 96-well round bottom plates, 104 CFSE-labeled target cells/well and a varying number of effector cells (in triplicate) were mixed, giving the indicated effector:target (E:T) ratios. Cultures were incubated for 4 h at 37°C in 5% CO2, and then propidium iodide (PI) was added to the samples and they were incubated for 5 min on ice. After PI staining, the samples were analyzed on a FACSCanto (BD) using the DIVA software (BD). Because cytolysis disrupts cell architecture, the frequency of PI-CFSE+ live target cells was recorded for each E:T and the relative sample lysis was calculated as 100 − % live [file pone.0219449.s004.pdf]
