# Supplementary material for: ICOS deficiency hampers the homeostasis, development and function of NK cells
Source: PLoS One. 2019 Jul 8;14(7):e0219449. doi: 10.1371/journal.pone.0219449 (PMC6613708; doi:10.1371/journal.pone.0219449)
Supplement: S1 File — The original western-blots for Fig 7 are shown. (PDF) [file pone.0219449.s005.pdf]

Original blots Figure 7C

Original blot for Phospho-Akt Thr 308

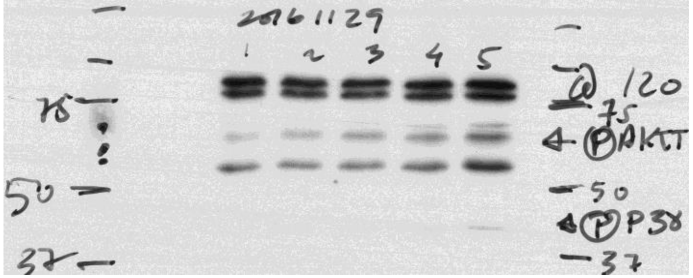

Original blot for Akt load

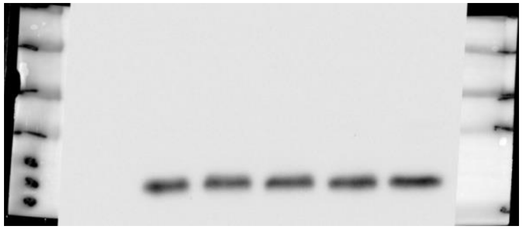

Original blots Figure 7F

Original blot for Phospho-P38

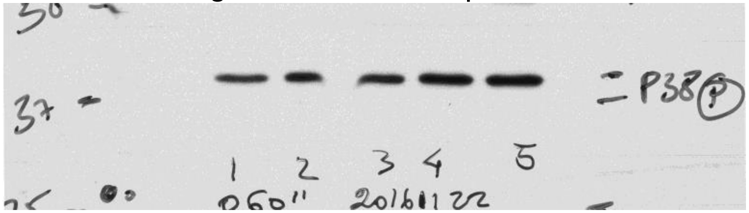

Original blot for P38 load

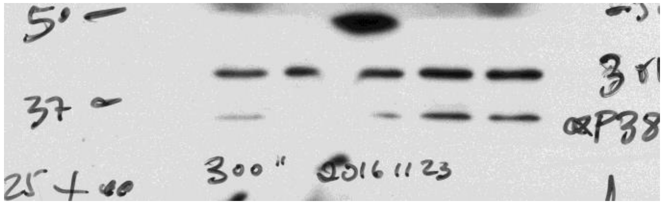

Original blots Figure 7D, E

Original blot for Phospho-Akt Phospho-Ser 473

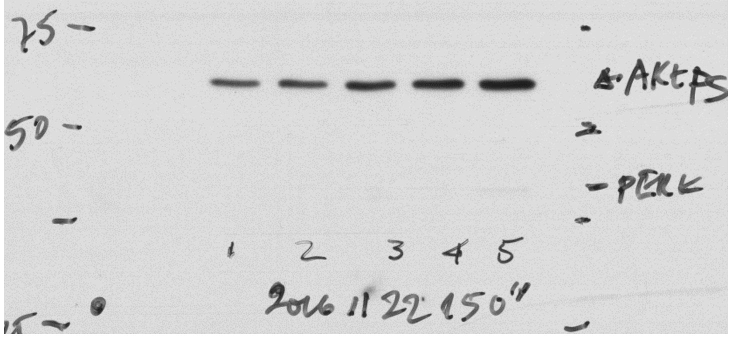

Original blot for Phospho-Erk

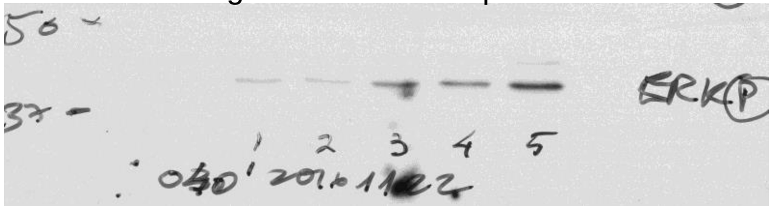

Original blot for Akt load (top) and Erk (bottom)

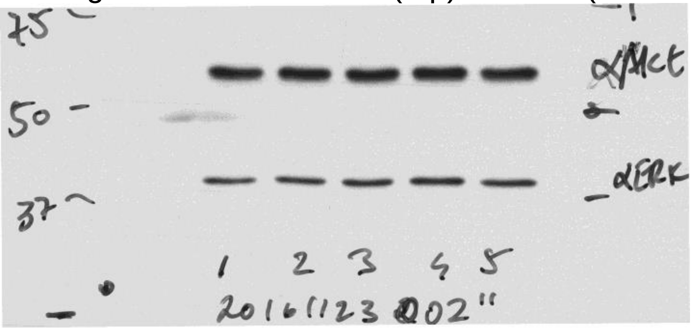

S1 File
